# T Cell Exhaustion and Dendritic Cell‐Mediated Tertiary Lymphoid Structures (TLSs) Modulation Affect Response to Neoadjuvant Chemoradiotherapy in Microsatellite Stable Rectal Cancer

**DOI:** 10.1002/advs.202514332

**Published:** 2025-11-05

**Authors:** Miao Wang, Jiejun Shi, Kai Xu, Leyi Yu, Xiaomao Yin, Jiexuan Wang, Lin Zhu, Xin Yang, Jingjing Qian, Wenqiang Wang, Liangchen Zhu, Xuan Dai, Zekun Zhao, Jinran Wu, Dongsheng Li, Zhiqian Hu, Qi Huang, Xinxing Li

**Affiliations:** ^1^ Department of General Surgery Tongji Hospital affiliated to Tongji University Frontier Science Center for Stem Cell Research School of Life Sciences and Technology Tongji University Shanghai 200092 China; ^2^ Department of General Surgery Tongji Hospital Medical College of Tongji University Shanghai 200092 China

**Keywords:** early exhausted T cells, locally advanced rectal cancer, neoadjuvant chemoradiotherapy, tertiary lymphoid structure

## Abstract

Locally advanced rectal cancer (LARC) presents significant treatment challenges, particularly in microsatellite stable (MSS) patients, who often show limited response to immunotherapy. In these cases, neoadjuvant chemoradiotherapy (neoCRT) followed by surgery remains the recommended approach. However, the response to neoCRT varies significantly among LARC patients. In this study, the role of the tumor microenvironment (TME) is explored, focusing on early‐stage exhausted T cells (early‐Tex) and tertiary lymphoid structures (TLS), in predicting neoCRT response in MSS LARC. Through multi‐omics analyses, it is found that immune features of the TME, rather than mutational status, are more closely associated with treatment response. Within the TME, it is observed that early‐Tex cells, a subset with both similarities and distinct differences compared to previously described precursor exhausted T (Tpex) cells, are enriched in responders and correlated with favorable treatment outcomes. Additionally, it is identified that TLSs are more abundant, activated, and mature in responders compared to non‐responders. LAMP3⁺ dendritic cells (DCs) play a pivotal role in suppressing TLS formation, with IRF8 as a key transcriptional regulator, which may ultimately affect therapeutic response. These findings suggest early‐Tex cells and modulation of TLS by LAMP3⁺ DCs can serve as indicators for optimizing neoCRT in MSS LARC.

## Graphical abstract

Using multi‐omics analysis, this study demonstrates that immune characteristics of the tumor microenvironment, rather than mutational profiles, are more strongly correlated with treatment response. Specifically, early exhausted T cells and tertiary lymphoid structures regulation by LAMP3⁺ dendritic cells emerge as key predictors of favorable outcomes after chemoradiotherapy, offering potential biomarkers to refine neoadjuvant chemoradiotherapy strategies in microsatellite stable Locally advanced rectal cancer patients.

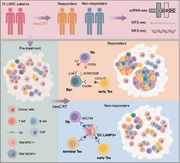

## 1 Introduction

Colorectal cancer (CRC) is the third most prevalent malignancy worldwide and the second leading cause of cancer‐related fatalitie.^[^ ^1^ ^]^ Approximately 30% of CRC are rectal cancer (RC).^[^ ^2^ ^]^ The incidence of RC is increasing annually, particularly among younger individuals under the age of 50.^[^ ^3^ ^]^ For various solid tumors, Immune checkpoint inhibitors (ICIs) have become the standard treatment,^[^ ^4^, ^5^, ^6^ ^]^ as well as in locally advanced RC (LARC) with microsatellite instability‐high (MSI‐H) or deficient mismatch repair (dMMR),^[^ ^7^ ^]^ but are largely ineffective in the majority (90–95%) of microsatellite stable (MSS) rectal cancers.^[^ ^8^ ^]^ In these cases, neoadjuvant chemoradiotherapy (neoCRT) followed by surgery remains the recommended treatment.^[^ ^9^ ^]^ The combination of preoperative neoCRT and surgery has significantly reduced the recurrence rate and improved the survival outcomes of LARCs.^[^ ^10^ ^]^ Despite its benefits, the response to neoCRT varies significantly among RC patients.^[^ ^11^ ^]^ A potential factor contributing to this limited efficacy is the complex and dynamic tumor microenvironment (TME), which plays a critical role in modulating therapeutic outcomes.^[^ ^12^ ^–^ ^14^ ^]^

Radiation therapy has been shown to induce immunogenic cancer cell death, promoting the release of tumor antigens and proinflammatory signals that stimulate innate and adaptive immune responses.^[^ ^15^ ^]^ However, TME comprises a heterogeneous mix of cellular components, including immune cells, stromal cells, and various extracellular matrix proteins, alongside non‐cellular elements such as cytokines and growth factors.^[^ ^16^, ^17^ ^]^ This intricate milieu shapes an immunosuppressive environment that can hinder the effectiveness of neoCRT.^[^ ^18^ ^]^ Advances in single‐cell RNA sequencing (scRNA‐seq) have enabled detailed characterization of TME complexity. Studies in esophageal adenocarcinoma and head and neck squamous cell carcinoma patients treated with neoCRT or neoadjuvant chemoimmunotherapy have revealed cellular and molecular features, such as CD103⁺CD8⁺ T cells, associated with treatment response.^[^ ^19^, ^20^ ^]^ In parallel, tertiary lymphoid structures (TLSs)—organized aggregates rich in T and B cells—have also been associated with improved responses to neoadjuvant immunotherapy across various cancer types.^[^ ^21^, ^22^ ^]^ Within this immune‐rich context, tumor‐reactive T cells play a central role in mediating cytotoxic effects. However, their effector functions could be curtailed by a broad spectrum of immunosuppressive mechanisms that are present in the TME.^[^ ^23^ ^]^ Notably, a distinct subset of dysfunctional T cells, termed progenitor or precursor exhausted T cells (Tpex), has garnered increasing attention. These Tpex cells exhibit remarkable proliferative capacity and possess the ability to differentiate into terminally exhausted T cells. Importantly, they demonstrate a preferential response to immunotherapy, suggesting their potential to play a pivotal role in modulating the TME.^[^ ^24^, ^25^, ^26^ ^]^

In CRC, neoCRT has been shown to enhance immune infiltration, including increased levels of activated CD8+ T cells^[^ ^27^ ^]^ and elevated expression of MHC‐I molecules,^[^ ^28^ ^]^ as demonstrated through immunohistochemistry and flow cytometry. However, despite these findings, few studies have leveraged scRNA‐seq to dissect the tumor microenvironment in LARC patients treated with neoCRT. And the potential involvement of key immune components—such as Tpex cells and TLSs—in shaping therapeutic response remains poorly elucidated and thus warrants further investigation.

In this study, we conducted whole exome sequencing (WES), whole transcriptome sequencing of bulk RNA (WTS), and scRNA‐seq on tumors from LARC patients who underwent neoCRT and surgery but exhibited varying responses. We found that differences in treatment response were closely associated with alterations in the tumor immune microenvironment, particularly the enrichment of Tpex and the formation of TLSs. The response group showed accumulated progenitor‐like Tex and TLS, as well as reduced immunosuppressive signals induced by mature dendritic cells (DCs), which collectively facilitated tumor cell apoptosis and suppressed tumor progression. These findings provide important evidence for optimizing neoadjuvant strategies for rectal cancer in the future.

## 2 Results

### 2.1 Immune‐Enriched TME, Rather than Mutations, Drives neoCRT Response of LARC

**Figure 1:**
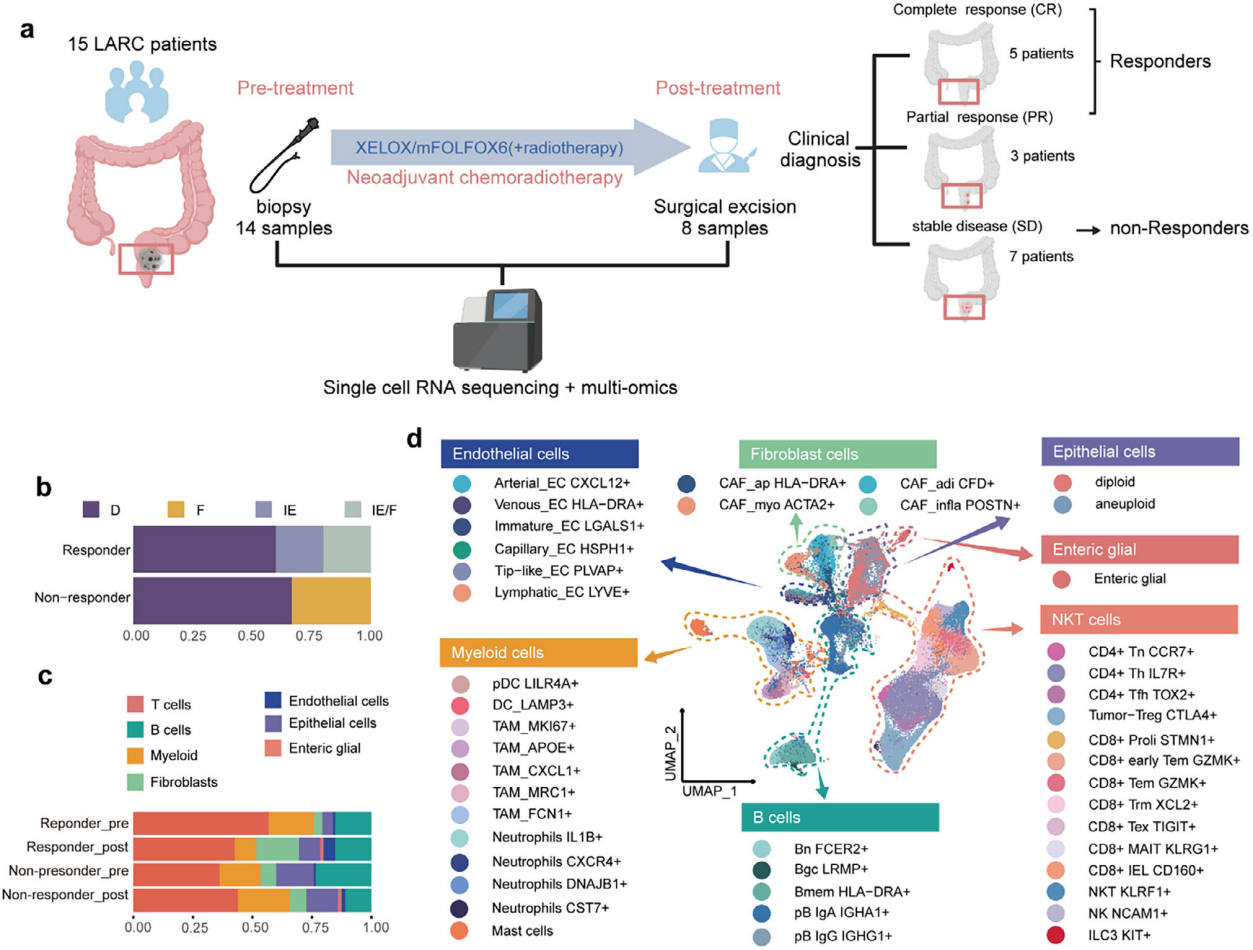
Analysis workflow and overview of tumor microenvironment changes induced by neoCRT. a) Overview of the study design. Created with BioGDP^[^ ^68^ ^]^ b) The distribution of tumor microenvironment subtypes identified in the previous study among responders and non‐responders before treatment. c) Relative proportions of major cell types in pre‐treatment and post‐treatment samples from responders and non‐responders. d) UMAP visualization of all cells, color‐coded by cell subtype.

To investigate the molecular basis underlying the efficacy of neoCRT, we performed multi‐omics analysis on fifteen patients with LARC (**Figures** **1**a; Figure S1a and Table S1, Supporting Information). Among these patients, five achieved a complete response (CR) and three achieved a partial response (PR), collectively classified as responders, while the remaining seven patients with stable disease (SD) were classified as non‐responders. Tumor tissues were collected via colonoscopy (pre‐treatment) and surgical resection (post‐treatment) for WES, WTS, and scRNA‐seq.

To evaluate whether mutational status correlates with treatment response, we compared the microsatellite instability (MSI) status of our patients with that of rectal adenocarcinoma (READ) patients from The Cancer Genome Atlas (TCGA) dataset. Among the 157 TCGA READ samples, six were classified as MSI, while the remaining samples were identified as MSS.^[^ ^29^ ^]^ Using the same cutoff (MSI score = 3.5), all patients in this study were classified as MSS (Figure S1b, Supporting Information). Furthermore, we identified high‐confidence somatic mutations, including those frequently observed in the TCGA READ cohort (e.g., *APC, TP53, KRAS*), as well as mutations associated with the Wnt signaling pathway, chromatin regulation, genome integrity, etc. Nearly all of these mutations were shared between responders and non‐responders (Figure S1c, Supporting Information). We also examined the copy number variation (CNV) status of these genes, which showed similar alteration patterns between responders and non‐responders (Figure S1d, Supporting Information). Additionally, we analyzed tumor mutational burden (TMB) and single‐nucleotide variant (SNV) profiles across the samples (Figure S1e–g, Supporting Information), which revealed no significant differences between responders and non‐responders. These findings suggest that somatic mutations are not the primary determinants of treatment efficacy.

To investigate the immune components of the TME in our samples, we applied a TME classification approach based on a transcriptomic framework that integrates cell‐type composition and the activity of key signaling pathways, inferred from gene expression profiles associated with specific cell types and biological processes.^[^ ^30^ ^]^ Transcriptomic data from seven pre‐treatment samples were integrated to explore the relationship between TME characteristics and response to neoCRT using this established TME signature.^[^ ^30^ ^]^ Each tumor sample was classified into one of four previously defined microenvironment subtypes:^[^ ^30^ ^]^ immune‐depleted (D), fibrotic (F), immune‐enriched (IE), and immune‐enriched/fibrotic (IE/F), based on their molecular functional profiles. The analysis revealed that responders exhibited microenvironment characteristics favorable for treatment even before therapy, as evidenced by the predominance of IE and IE/F subtypes (Figure 1b). These findings underscore the pivotal role of the TME in the neoCRT treatment process and its significant impact on therapeutic outcomes.

### 2.2 Overview of TME Remodeling by neoCRT

To investigate changes in the TME of LARC patients, we integrated single‐cell transcriptomic data collected pre‐ and post‐neoCRT treatment. After filtering out doublets and low‐quality cells, we retained a total of 131285 cells for analysis and performed unsupervised clustering. Seven major cell types were identified based on canonical marker gene expression (Figure 1c,d; Figure S2a,b, Supporting Information), including T cells, B cells, myeloid cells, fibroblasts, epithelial cells, endothelial cells, and enteric glial cells.

Malignant epithelial cells were identified by detecting copy number variation (CNV) patterns within the epithelial cell population (Figure S2c, Supporting Information). A marked post‐treatment reduction of malignant cells (aneuploid) was observed in responders but not in non‐responders, highlighting the differences in treatment efficacy between the two groups (Figure S2d,e, Supporting Information). Gene set variation analysis (GSVA) of malignant cells revealed distinct pathway activities between responders and non‐responders. In responders, pathways associated with DNA damage and apoptosis were significantly activated after treatment, whereas epithelial‐to‐mesenchymal transition (EMT)‐related pathways were suppressed. In contrast, EMT‐related pathways were upregulated in non‐responders post‐treatment (Figure S2f, Supporting Information). These findings confirmed that neoCRT effectively induces DNA damage to restrict or eradicate the proliferation and migration of cancer cells.^[^ ^31^ ^]^

Furthermore, interferon‐gamma and Fas‐signaling were upregulated after neoCRT in responders but suppressed in non‐responders (Figure S2f, Supporting Information), underscoring the critical role of immune components in driving favorable outcomes.^[^ ^32^ ^]^ Consistently, neoCRT induced significant alterations in the composition and abundance of other cell types within the TME (Figure 1c; Figure S2g, Supporting Information). Fibroblasts expanded substantially post‐treatment, particularly in responders. Meanwhile, T cells and myeloid cells showed reduced abundance, suggesting their potential involvement in treatment outcomes.

### 2.3 neoCRT Promotes Fibrosis by Modulating Fibroblasts and Macrophages

Although radiotherapy provides significant benefits, long‐term fibrosis can severely impact patients’ quality of life, with fibroblasts playing a critical role.^[^ ^33^ ^]^ We focused on cancer‐associated fibroblasts (CAFs) and identified four subtypes (Figure S3a,b, Supporting Information): myofibroblast‐like CAFs (CAF_myo, ACTA2+), adipose CAFs (CAF_adi, CFD+), inflammatory CAFs (CAF_infla, POSTIN+), and antigen‐presenting CAFs (CAF_ap, HLA‐DRA+). Among these, CAF_myo, CAF_adi, and CAF_infla increased after treatment, while CAF_ap decreased (Figure S3c,d, Supporting Information).

To explore the differentiation process of CAFs, we reconstructed their differentiation trajectories and identified two distinct paths. One trajectory (Cell Fate 1, CF1) was primarily composed of CAF_adi and CAF_infla, while the other (Cell Fate 2, CF2) was dominated by CAF_myo (Figure S3e, Supporting Information). Both branches activated fibrosis‐ and extracellular matrix (ECM)‐related pathways. And CF1 trajectory also upregulated T cell activation pathways (Figure S3f, Supporting Information) and increased after treatment (Figure S3g, Supporting Information), suggesting their involvement in anti‐tumor immunity. Furthermore, cells in both trajectories were enriched in post‐treatment samples (Figure S3h, Supporting Information), indicating that neoCRT induced CAF differentiation and promoted fibrosis. In responders, CAFs were more likely to differentiate into the CAF_infla subtype, which supports anti‐tumor effects (Figure S3c, Supporting Information), consistent with previous findings.^[^ ^34^ ^]^

In addition to pro‐fibrotic cells native to organs, other cell types, such as tumor‐associated macrophages (TAMs), also significantly contribute to fibrosis.^[^ ^35^, ^36^ ^]^ By re‐clustering myeloid cells, we identified five TAM subtypes (Figure S4a,b, Supporting Information): TAM MKI67+ (proliferative), TAM APOE+, TAM CXCL1+, TAM MRC1+, and TAM VEGFA+. Trajectory analysis revealed two distinct differentiation pathways originating from proliferative TAM MKI67+ cells: one toward TAM MRC1+ and the other toward TAM VEGFA+ (Figure S4c, Supporting Information). Traditional “classically activated” (M1) and “alternatively activated” (M2) macrophage markers (Table S2, Supporting Information)^[^ ^37^, ^38^ ^]^ could not fully explain the observed TAM subtypes in this study (Figure S4d, Supporting Information). Functional analysis revealed that TAM VEGFA+ was enriched in functions related to angiogenesis, extracellular matrix interaction, while TAM MRC1+ exhibited prominent roles in macrophage activation and fibroblast proliferation (Figure S4e, Supporting Information). These findings suggest that TAMs may play a key role in modulating fibroblast behavior.

To further investigate TAM‐CAF interactions, we analyzed cell‐cell communication and found fibrosis‐related ligand‐receptor (TGFB‐TGFBR,^[^ ^39^, ^40^ ^]^ PDGF‐PDGFR^[^ ^41^ ^]^) were significantly enriched in both TAM MRC1+ and TAM VEGFA+ subtypes, with signaling levels elevated post‐treatment (Figure S4f, Supporting Information). Interestingly, the ligand‐receptor pair OSM‐OSMR, known to regulate fibroblast proliferation and fibrosis,^[^ ^42^ ^]^ was decreased in responders after neoCRT but increased in non‐responders, associated with neoCRT response (Figure S4f, Supporting Information).

These observations indicate that neoCRT may induce two distinct TME response modes. In responders, CAFs preferentially differentiate into the inflammatory CAF (CAF_infla) subtype, which upregulates T‐cell activation pathways, reflecting an “immune‐enhanced” TME. In contrast, in non‐responders, TAM‐mediated OSM–OSMR signaling is elevated, promoting fibroblast proliferation and fibrosis, indicative of a “fibrosis‐enhanced” TME. Collectively, the increase of CAF_infla in responders and the enhanced TAM OSM–OSMR signaling in non‐responders highlight the interplay between immune activation and fibrotic remodeling in shaping treatment outcomes.

### 2.4 Proliferative Early‐Tex and T Cell Activation Drive neoCRT Efficacy

**Figure 2:**
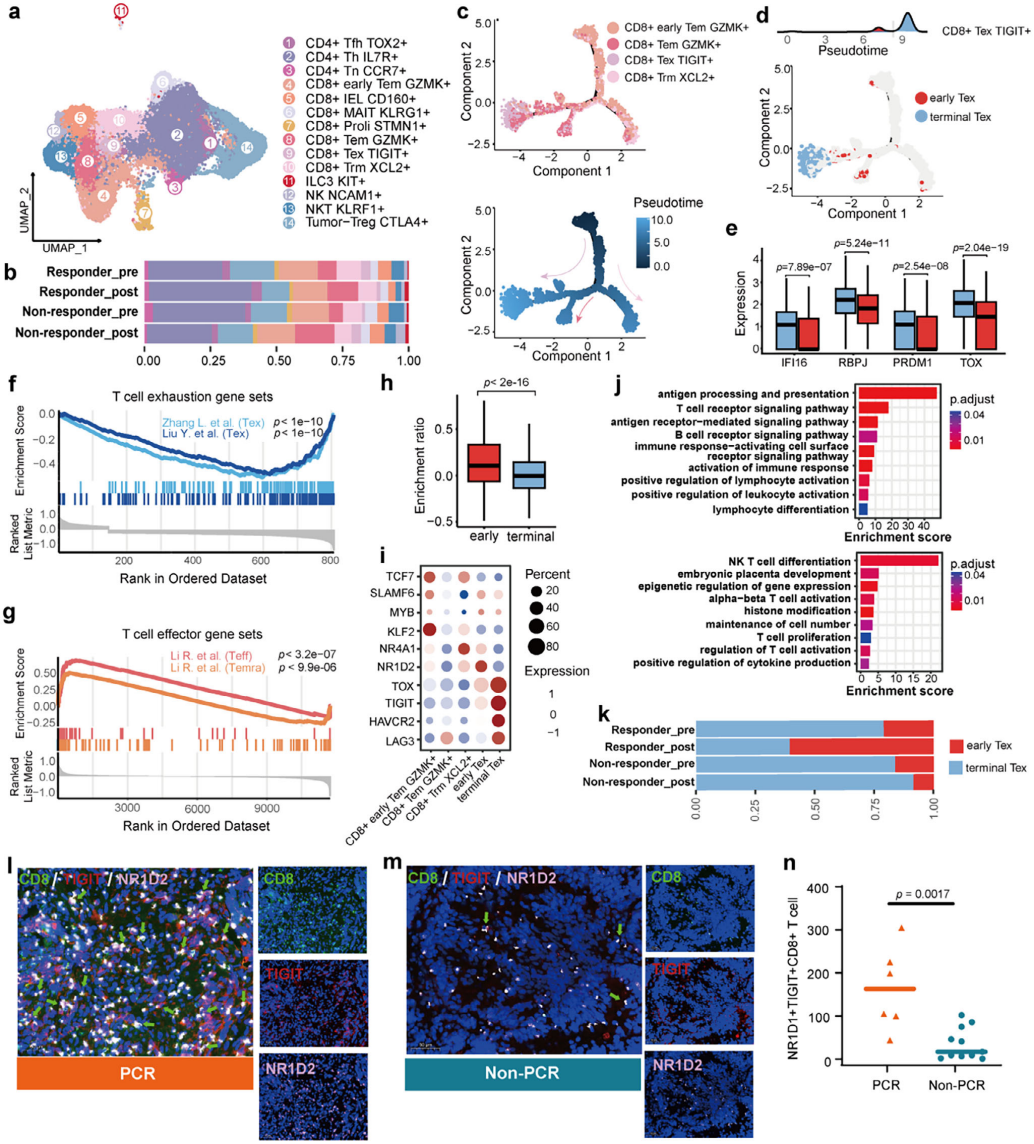
Early‐Tex with immune activity increased in responders after neoCRT. a) UMAP of T cell sub‐clusters. b) Relative proportions of T cell sub‐types in pre‐treatment and post‐treatment samples from responders and non‐responders. c) Developmental trajectory of CD8+ T cells. Colored by cell types (top) and pseudotime (bottom). Arrows in the bottom plot denote potential differentiation paths. d) Density of CD8+ TIGIT+ Tex cells along the developmental trajectory (top) and the positioning of early‐Tex and terminal‐Tex on the trajectory (bottom). e) Expression levels of exhaustion‐related genes in early‐Tex vs terminal‐Tex. Statistical significance was determined using the Wilcoxon test. f) Gene set enrichment analysis (GSEA) of T cell exhaustion signatures using genes differentially expressed between early Tex and terminal Tex. Two published Tex‐related gene sets, Zhang L. et al. and Liu Y. et al., were used for evaluation. g) Gene set enrichment analysis (GSEA) of T cell effector signatures using genes differentially expressed between early Tex and terminal Tex. The effector gene sets were derived from Li R. et al., representing effector T cell (Teff) and activated effector memory T cells (Temra) signatures. h) Boxplot showing the ratio of gene signature scores for progenitor vs terminal exhausted Tex in early‐Tex and terminal‐Tex. Statistical significance was determined using the two‐tailed *t*‐test. i) Expression of differentially expressed genes (DEGs) between early and terminal‐Tex. j) Gene Ontology (GO) terms enriched in genes upregulated in early‐Tex from responders vs non‐responders before treatment (top) and genes upregulated after treatment vs before treatment in responders (bottom). Benjamini–Hochberg adjusted *p*‐value< 0.05. k) Relative proportions of early‐Tex and terminal‐Tex in pre‐treatment and post‐treatment samples from responders and non‐responders. l) Immunofluorescence staining of CD8, TIGIT, and NR1D2 in tumor tissue with pathological complete response to neoCRT. Arrows indicate early‐Tex. m) Immunofluorescence staining of CD8, TIGIT, and NR1D2 in tumor tissue with pathological non‐response to neoCRT. Arrows indicate early‐Tex. n) Comparison of the number of early‐Tex in tumor tissues with the pathological complete response and non‐response/partial response group. Statistical significance was determined using the two‐tailed *t*‐test.

Building on the observation that a certain CAF subset may contribute to T cell activation (Figure S3f, Supporting Information), we next examined the T cell compartment to determine how T cell states influence neoCRT efficacy. By comparing the changes in the proportion of T cell subtypes before and after treatment, we found that effector memory T cells (Tem) increased in both responders and non‐responders after neoCRT. In responders, regulatory T cells (Tumor‐Treg) and exhausted T cells (Tex) decreased, while naive T cells (Tn), helper T cells (Th), and follicular helper T cells (Tfh) increased. In contrast, Tumor‐Treg and Tex increased in non‐responders, while Tn and Th decreased (**Figure** **2**a,b; Figure S5a,b, Supporting Information).

We reconstructed the differentiation trajectories of CD4+ and CD8+ T cells (Figure 2c; Figure S5c, Supporting Information). CD4+ T cells originated from Tn and differentiated into two distinct branches: Th, with upregulated genes associated with positive regulation of lymphocyte activation and immune response, and Treg cells, characterized by genes linked to negative immune regulation (Figure S5c,d, Supporting Information). Notably, cells within the Treg trajectory decreased in responders and increased in non‐responders after treatment (Figure S5e, Supporting Information), which correlated with therapeutic efficacy.

For CD8+ T cells, early Tem cells differentiated into three trajectories, ultimately forming Tex, Tem, and tissue‐resident memory T cells (Trm). Intriguingly, the pseudotime distribution of Tex showed two peaks, indicating two distinct subtypes (Figure 2d). To explore whether there are Tpex‐like cells that exert their function during the neoCRT, we divided Tex into early‐Tex and terminal‐Tex based on pseudotime inference (Figure 2d). Compared to early‐Tex, terminal‐Tex showed higher expression of exhaustion‐related genes such as *TOX*, *RBPJ*, and *PRDM1* and co‐inhibitory molecules like *PDCD1*, *CTLA4*, and *LAG3* (Figure 2e; Figure S5f, Supporting Information), as well as activation of exhaustion‐promoting transcription factors like *BATF* ^[^ ^43^ ^]^ and *ETS1* ^[^ ^44^ ^]^ (Figure S5g, Supporting Information). Consistently, Gene sets associated with T cell exhaustion derived from published studies (Zhang L. et al. and Liu Y. et al.)^[^ ^45^, ^46^ ^]^ were also significantly enriched in terminal‐Tex (Figure 2f). In contrast, early Tex cells showed enrichment of effector‐related gene sets derived from Li R. et al. (Figure 2g),^[^ ^47^ ^]^ including signatures of effector T cells (Teff) and activated effector memory T cells (Temra), as well as pathways involved in cytokine production and proliferation (Figure S5h, Supporting Information), highlighting their proliferative capacity and anti‐tumor potential. To assess whether early‐Tex exhibited a Tpex phenotype described in previous studies,^[^ ^48^ ^]^ we scored cells using progenitor and terminal Tex gene signatures (Table S2, Supporting Information). Early‐Tex demonstrated significantly higher transcriptional similarity to Tpex (Figure 2h), supporting their progenitor‐like state.

We further examined the expression of previously identified key factors for Tpex, including *TCF7*, *SLAMF6*, and *MYB*, which were originally characterized in infection and melanoma models.^[^ ^49^, ^50^, ^51^ ^]^ However, they did not specifically distinguish early‐Tex from terminal‐Tex (Figure 2i), differing from the classical Tpex population identified within the TME under immunotherapy.^[^ ^49^, ^50^ ^]^ To gain deeper insights, we performed differential gene expression analysis between early‐Tex and terminal‐Tex, and found that early‐Tex highly expressed *NR1D2*(Figure 2i), a transcription factor associated with cell proliferation and motility.^[^ ^52^ ^]^ Target gene analysis of *NR1D2* showed enrichment in pathways related to cell proliferation, like “mitotic nuclear division” and “Adhesion‐dependent cell spreading” (Figure S5i, Supporting Information), indicating its potential role in maintaining the proliferative capacity of early‐Tex. Notably, NR1D2+ Tex showed greater activation in responders than in non‐responders before treatment, and further upregulated after treatment(Figure 2j). Furthermore, responders exhibited a higher proportion of early‐Tex after treatment (Figure 2k). In line with these findings, we performed multiplex immunofluorescence (mIF) on an additional cohort of 17 tumor samples collected before neoCRT, using *CD8*, *TIGIT*, and *NR1D2* as markers to identify early‐Tex (Figure 2l,m). The results showed a greater accumulation of early‐Tex in tumor tissues with pathological complete response to neoCRT (Figure 2l–n), suggesting that NR1D2+ Tex is associated with better clinical outcomes in LARC.

### 2.5 neoCRT Facilitates the Formation and Maturation of Tertiary Lymphoid Structures (TLSs)

**Figure 3:**
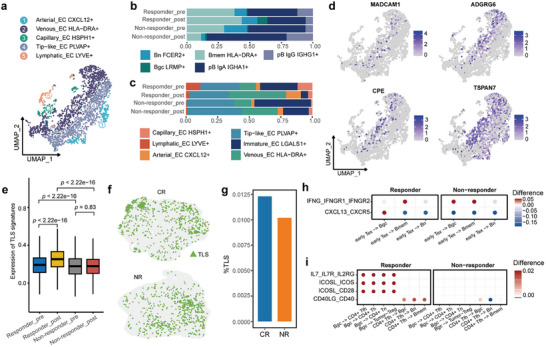
neoCRT promotes TLS formation. a) UMAP of endothelial cell sub‐clusters. b,c) Relative proportions of B cells (b) and endothelial cells (c) in pre‐treatment and post‐treatment samples from responders and non‐responders. d) expression of high endothelial venule (HEV) marker genes. e) TLS signature scores across four groups: pre‐treatment and post‐treatment samples from responders and non‐responders. Statistical significance was determined using the Wilcoxon test. f) Spatial distribution of identified TLSs in a complete response (CR) sample (top) and a non‐response (NR) sample (bottom). g) TLS proportion in CR and NR. h,i) Altered ligand‐receptor (L‐R) pair signaling between early‐Tex cells and B cells (h) and between CD4+ T cells and B cells (i). The color of the dots indicates the difference in communication probability between post‐treatment and pre‐treatment.

TLSs are organized aggregates of immune cells that arise in non‐lymphoid tissues under pathological conditions and are generally associated with a favorable prognosis.^[^ ^53^, ^54^ ^]^ Previous studies have established that Tfh cells, Bgc cells, and HEVs are integral components of TLSs and play crucial roles in their formation.^[^ ^55^, ^56^, ^57^, ^58^ ^]^ In this study, Tfh cells were elevated in responders post‐treatment (Figure 2b). Additionally, re‐clustering of B cells (Figure S6a,b, Supporting Information) and endothelial cells (**Figure** **3**a; Figure S6c, Supporting Information) revealed a substantial increase in germinal center B cells (Bgc, LRMP+) (Figure 3b; Figure S6d, Supporting Information) and venous endothelial cells (Venous EC, HLA‐DRA+) (Figure 3c; Figure S6e, Supporting Information) in responders post‐treatment, and Venous EC exhibited high expression of high endothelial venules (HEV) markers (Figure 3d). Together, these findings suggest that neoCRT may facilitate TLS formation.

To investigate this hypothesis, we collected TLS‐related gene signatures identified in previous studies^[^ ^54^, ^59^, ^60^, ^61^ ^]^ and focused on genes that were significantly differentially expressed before and after treatment, which we defined as neoCRT‐related TLS signatures (Table S3, Supporting Information). By comparing the expression levels of these TLS signatures in responders and non‐responders before and after treatment, we observed a significant increase in responders, whereas non‐responders showed no notable changes (Figure 3e). We further analyzed published spatial transcriptomic data from rectal cancer patients,^[^ ^34^ ^]^ including responder (CR) and non‐responder (NR). TLS regions were defined as capture spots that exhibited high expression levels of both T‐cell and B‐cell signatures (Figure 3f), and TLS signature genes were significantly enriched in these regions (Figure S6f, Supporting Information). Notably, CR demonstrated a higher abundance of TLS compared to NR (Figure 3g). These findings provide strong evidence that neoCRT promotes TLS formation, underscoring its potential role in modulating the tumor microenvironment.

To further examine differences in TLSs between responders and non‐responders, we performed differential expression analysis of TLS regions from both groups. Gene Ontology (GO) analysis revealed that genes upregulated in responders were enriched in terms related to immune response, lymphoid cell proliferation, and lymphoid cell activation (Figure S6g, Supporting Information), suggesting that TLSs in responders exhibit greater activation. Additionally, trajectory analysis of TLS development indicated that TLSs in non‐responders were skewed toward earlier pseudotime, while those in responders were biased toward later pseudotime values (Figure S6h, Supporting Information). Genes associated with TLS formation and maturation, such as *CXCR4*, *CCL21*, *CCL19*, and *CXCL12* ^[^ ^62^, ^63^ ^]^ were upregulated along this trajectory (Figure S6i, Supporting Information). These results indicate that neoCRT promotes the formation of more activated and mature TLSs in responders compared to non‐responders.

Finally, analysis of TLS‐related intercellular interactions revealed upregulated ligand‐receptor pairs, such as IFNG‐IFNGR and CXCL13‐CXCR5, between early‐Tex and B cells, which are critical for B cell activation and recruitment (Figure 3h). Additionally, interactions between Tfh cells and B cells, including IL7‐IL7R, ICOSL‐CD28, and CD40LG‐CD40, were enhanced in responders after neoCRT, promoting T and B cell activation. In contrast, these interactions were diminished in non‐responders (Figure 3i), further supporting the conclusion that neoCRT facilitates TLS formation in responders.

### 2.6 DC LAMP3+ Inhibits TLS Formation via T Cell Exhaustion

**Figure 4:**
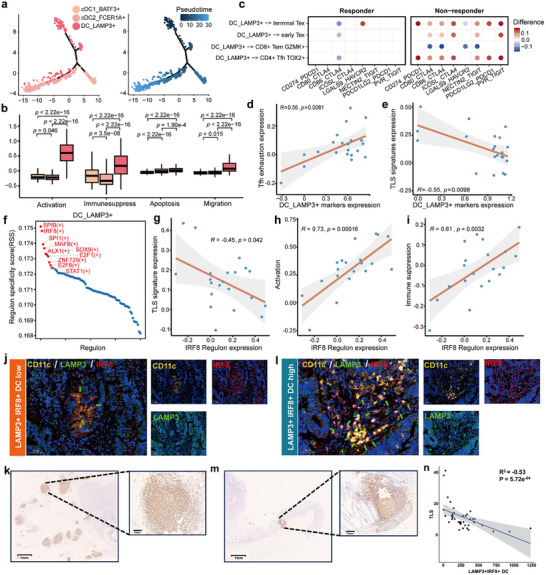
DC LAMP3+ inhibited TLS formation via promoting T cell exhaustion. a) Developmental trajectory of dendritic cells (DCs). Colored by sub‐cell types (left) and pseudotime (right). Arrows in the right plot denote potential differentiation paths. b) Signature scores of activation, immune‐suppression, apoptosis, and migration related genes across DC subsets. Statistical significance was determined using the Wilcoxon test. c) Altered ligand‐receptor (L‐R) pair signaling from DC_LAMP3+ to T cells. Dot colors indicate the difference in communication probability between post‐treatment and pre‐treatment. d) Scatterplot showing the Pearson correlation between the expression of DC_LAMP3+ marker genes in DC_LAMP3+ and the expression of exhaustion signatures in T follicular helper (Tfh) cells. e) Scatterplot presents the Pearson correlation between the expression of TLS signatures and the expression of DC_LAMP3+ marker genes in DC_LAMP3+. f) Top 10 specific transcription factor (TF) regulons identified in DC_LAMP3+. g–i) Scatterplots present the Pearson correlation between expression of the IRF8 regulon and the expression of TLS signatures(g), activation signatures (h), and immune suppression signatures (i) in DC_LAMP3+. j) Multiplex immunofluorescence staining of CD11c, LAMP3 and IRF8 in tumor tissue with a low abundance of LAMP3+ IRF8 + DCs. Arrows indicate LAMP3+ IRF8+ DC cells. k) The distribution of TLSs in tumor tissue with a low abundance of LAMP3+ IRF8 + DCs. l) Multiplex immunofluorescence staining of CD11c, LAMP3 and IRF8 in tumor tissue with a high abundance of LAMP3+ IRF8 + DCs. Arrows indicate LAMP3+ IRF8+ DC cells. m) The distribution of TLSs in tumor tissue with a high abundance of LAMP3+ IRF8 + DCs. n) Scatterplot showing the Pearson correlation between the number of LAMP3+ IRF8+ DC cells and the abundance of TLSs.

In addition to T and B cells, DCs are key components of TLSs. Based on known marker genes, we identified three DC subsets: DC_BATF3+, DC_FCER1A+, and DC_LAMP3+ (Figure S4a, Supporting Information). DC_BATF3+ were classified as classical cDC1 based on the expression of *BATF3*, *XCR1*, and *CLEC9A*, while DC_FCR1A+ were identified as cDC2 due to their high expression of *CD1C*, *CD1E*, and *FCER1A*.^[^ ^64^ ^]^ Previous studies have shown that LAMP3+ DCs are the most active DC subset in tumors, characterized by high migratory potential, and are likely derived from both cDC1 and cDC2 lineages.^[^ ^65^, ^66^ ^]^ Using Monocle2, we reconstructed the developmental trajectory of these DC subsets and found that DC_LAMP3+ can differentiate from both DC_BATF3+ and DC_FCER1A+ (**Figure** **4**a). We further analyzed the functional characteristics of these DC subsets, including their activation, apoptosis, immunosuppressive, and migratory capacities. Among them, DC_LAMP3+ exhibited the highest scores in all aspects compared to the other subsets (Figure 4b). Given the activation and immunosuppressive roles of DC_LAMP3+, we investigated their interactions with other cell types. DC_LAMP3+ interacted with Tfh cells, early‐Tex, and terminal‐Tex through co‐inhibitory molecule interactions, including PDCD1LG2‐PDCD1, NECTIN2‐TIGIT, and LGALS9‐HAVCR2 (Figure 4c). Notably, these interaction signals were reduced in responders but increased in non‐responders, suggesting that DC_LAMP3+ may promote Tfh exhaustion, an effect that is mitigated in responders after treatment.

Building on these findings, we hypothesized that DC_LAMP3+ may influence TLS formation by driving T cell exhaustion. To test this, we examined the correlation between DC_LAMP3+, Tfh exhaustion, and TLS formation. Marker genes of DC_LAMP3+ were positively correlated with exhaustion‐related genes (Figure 4d) and negatively correlated with TLS signature genes (Figure 4e). These results suggest that DC_LAMP3+ may inhibit TLS formation by promoting Tfh exhaustion, a process suppressed in responders, thereby enhancing anti‐tumor immunity.

To further investigate the regulatory mechanisms underlying the immunosuppressive functions of DC_LAMP3+, we applied SCENIC^[^ ^67^ ^]^ to identify transcription factors potentially driving these functions. Using the Regulon Specificity Score (RSS), we identified specific regulons in DC_LAMP3+ (Figure 4f). Among these, the IRF8 regulon exhibited decreased activity in responders after treatment but was upregulated in non‐responders (Figure S7a, Supporting Information). A similar pattern was observed for *IRF8* expression in WTS data (Figure S7b, Supporting Information), indicating a potential role for *IRF8* during treatment. Furthermore, we found a significant negative correlation between the IRF8 regulon and TLS formation (Figure 4g). In contrast, the IRF8 regulon was positively correlated with DC activation and immunosuppressive capacity (Figure 4h,i). These findings suggest that the IRF8 regulon plays a critical regulatory role in the functional development of DC_LAMP3+. To further validate the association between TLSs and LAMP3+ DCs, we retrospectively analyzed postoperative tumor samples from 40 patients with LARC (Table S5, Supporting Information) using multiplex immunohistochemistry (IHC) and mIF. Our findings revealed a higher abundance of TLSs in tumor tissue with a low abundance of LAMP3+ IRF8 + DCs (Figure 4j,k) compared to tumor tissue with a high abundance of LAMP3+ IRF8 + DCs (Figure 4l,m). We observed a significant negative correlation between CD11c+ LAMP3+ IRF8+ DCs and TLS density (Figure 4n), reinforcing the notion that LAMP3+ DCs contribute to TLS suppression and exert immunosuppressive effects within the tumor microenvironment, with *IRF8* playing a key regulatory role in this process.

## 3 Discussion

As the majority of LARC cases are MSS, neoCRT followed by surgery remains the recommended standard treatment. However, few studies have explored the TME of LARC at single‐cell resolution following neoCRT treatment. Here, we identified key immune microenvironment features that influence the response to neoCRT in MSS LARC. Specifically, we observed a distinct population of early‐stage exhausted T cells (early‐Tex) and discovered that DC LAMP3+ cells modulate TLS, which together impact the treatment response. Our findings suggest that immune features, rather than mutational status, play a more substantial role in determining the effectiveness of neoCRT in MSS LARC.

**Figure 5:**
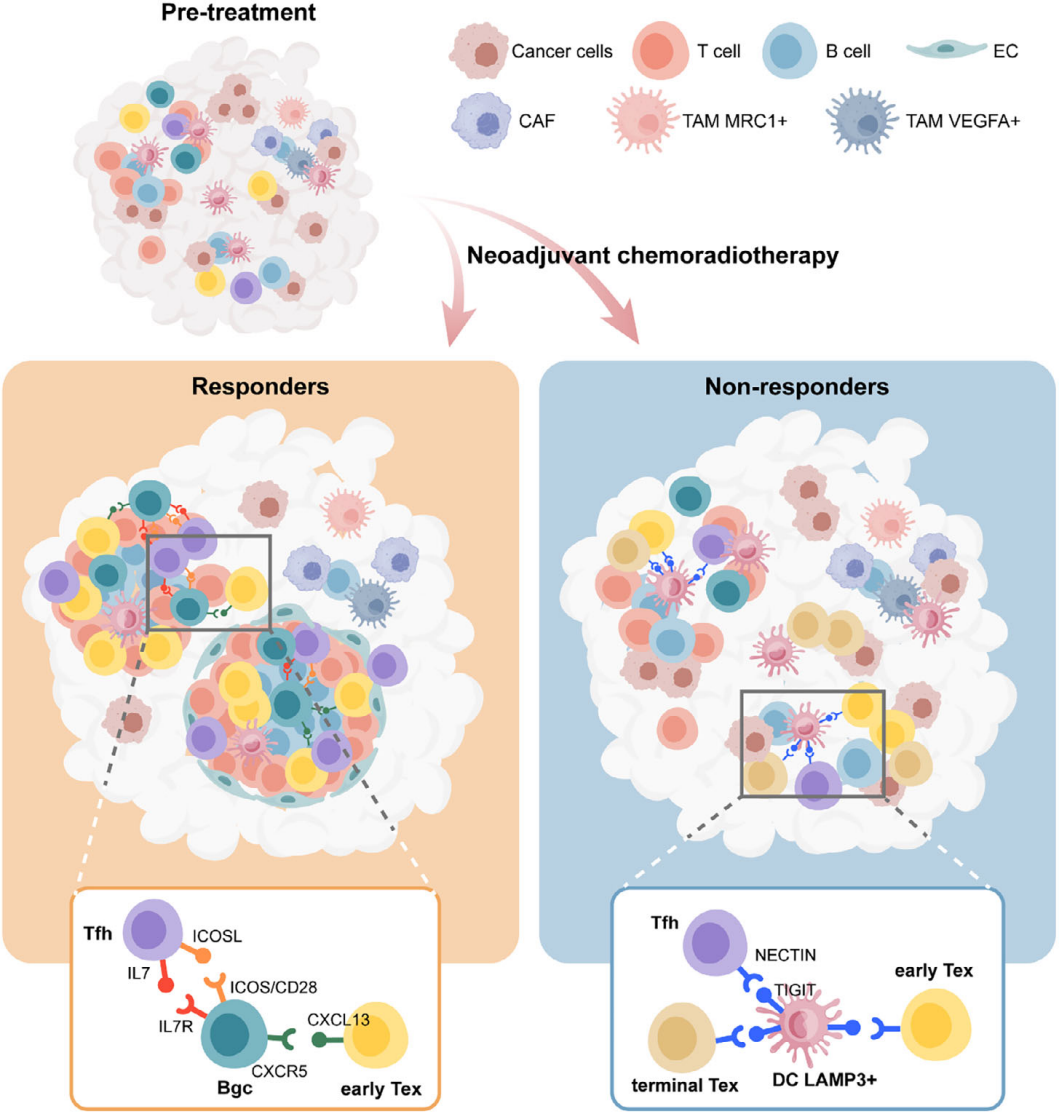
The local immunity model underlying neoCRT in LARC. In responding patients, neoCRT establishes a treatment‐favorable tumor microenvironment (TME), characterized by chemoradiotherapy‐induced tumor cell death, enhanced interactions between T follicular helper (Tfh) cells, early exhausted T cells (early‐Tex), and B cells, which promote B cell activation, migration, and tertiary lymphoid structure (TLS) formation. In contrast, in non‐responding patients, DC_LAMP3+ induces T cell exhaustion, suppresses TLS formation, and contributes to resistance to neoCRT. Created with BioGDP.^[^ ^68^ ^]^

The early‐Tex identified in this study shares some characteristics with previously described Tpex cells. However, unlike Tpex cells, which have been associated with immune memory and resilience in various contexts, early‐Tex cells have not been extensively studied in the context of neoCRT. Our study highlights the role of the transcription factor *NR1D2* in early‐Tex cells, which appears to play a crucial role in maintaining their proliferative capacity. Additionally, our data show that established Tpex marker genes, such as *TCF7* and *MYB*, do not effectively distinguish early‐Tex cells in our cohort. This further supports the notion that early‐Tex cells represent a distinct subset of exhausted T cells, differing from Tpex cells in both molecular signature and function. Furthermore, we identified potential crosstalk between early‐Tex and Bgc cells mediated through IFNG‐IFNGR and CXCL13‐CXCR5 ligand‐receptor axes, which may facilitate Bgc cell activation and recruitment (**Figure** **5**). These findings suggest that early‐Tex cells may have unique roles in the immune response to neoCRT, warranting further investigation into their therapeutic potential.

TLS has been implicated in promoting anti‐tumor immunity; however, its regulation remains incompletely understood. In this study, we observed a higher abundance of TLSs in responders, which were also more activated and mature compared to those in non‐responders (Figure 5). Moreover, we identified DC LAMP3+ cells—the most active dendritic cell subset within tumors—as negative regulators of TLS formation in MSS LARC. These cells interacted with Tfh cells and early‐Tex via co‐inhibitory molecules, which contributed to the exhaustion of T cells and accumulation of terminal‐Tex cells in non‐responders (Figure 5). And *IRF8*, a transcription factor associated with dendritic cell differentiation and maturation, played a critical role in this process. This immunosuppressive effect appeared to profoundly impact the anti‐tumor immune response to neoCRT. The modulation of TLS by DC LAMP3+ cells suggests a complex regulatory mechanism in which dendritic cells not only function to present antigens but also influence the formation of structures that might aid in initiating or enhancing immune responses. This finding expands our understanding of how DCs contribute to shaping the TME and suggests that DC‐mediated suppression of TLS may represent an important mechanism for immune evasion in MSS rectal cancer.

Additionally, exploring the broader immune landscape of MSS rectal cancer through techniques such as single‐cell RNA sequencing could provide a more comprehensive understanding of the immune cells and molecular pathways involved in therapy resistance and response. Investigating other immune cell subsets, including Tregs and myeloid‐derived suppressor cells (MDSCs), could reveal additional targets for therapeutic intervention.

## 4 Conclusion

In conclusion, our study highlights the importance of immune microenvironment features in influencing the response to neoCRT in MSS LARC. The discovery of early‐Tex cells and the regulatory role of DC LAMP3+ cells in TLS modulation expands our understanding of immune exhaustion and tumor immunity in MSS rectal cancer. These findings lay the foundation for further investigation into immune‐based therapies and personalized treatment strategies aimed at improving outcomes for patients with MSS LARC.

## 5 Experimental Section

### Sample Collection

This study enrolled 15 patients (9 males and 6 females; median age 58 years, range 40–76) diagnosed with locally advanced rectal cancer at Tongji Hospital between July 2022 and May 2023. Eligibility criteria required both metastasis‐free status (confirmed by baseline imaging) and no prior history of oncological treatments, including radiotherapy or chemotherapy. Patients with secondary malignancies were excluded from the analysis. All patients completed neoadjuvant chemoradiotherapy followed by curative‐intent surgical resection. Rectal cancer biopsy specimens were obtained via colonoscopy before neoCRT, and collected tumor tissue samples again from the rectal lesion at the time of surgical resection following completion of neoCRT. An additional 40 LARC samples were collected for IHC/mIF analysis. The patients had a median age of 69.5 years (IQR, 60.5–73.0), 57.5% were male, and all were metastasis‐free.

### Participant Details and Clinical Treatment

Fifteen patients enrolled in this study completed neoadjuvant chemoradiotherapy followed by curative‐intent surgical resection. The neoadjuvant treatment protocol comprised sequential chemoradiotherapy followed by consolidation chemotherapy, structured as follows:

1. Induction Chemotherapy: A single cycle of the XELOX regimen was administered. Oxaliplatin (130 mg m^−^^2^) was delivered intravenously on day 1 (D1), while capecitabine (1000 mg m^−^^2^) was administered orally twice daily from day 1–14 (D1‐14).
2. Concurrent Chemoradiotherapy: Following a 1 week washout period after induction chemotherapy, radiotherapy was initiated. Intensity‐modulated radiation therapy (IMRT) was employed to deliver a total dose of 50.4 Gy in 28 fractions (1.8 Gy per fraction, 5 fractions weekly) targeting the primary tumor and regional lymph nodes. Concurrent oral capecitabine was maintained at the same dosage and schedule as in the induction phase.
3. Consolidation Chemotherapy: Two additional cycles of the XELOX regimen commenced 21 days post‐radiotherapy. Each cycle consisted of oxaliplatin (130 mg m^−^^2^, intravenous, D1) and capecitabine (1000 mg m^−^^2^ bid, oral, D1‐14), followed by a 7‐day treatment‐free interval (D15‐21), with a 21‐day cycle duration.
4. Surgical Intervention: Total mesorectal excision (TME) was performed 2 weeks after the final chemotherapy cycle to optimize pathological complete response (pCR). The timing of surgery was determined based on radiobiological principles to maximize tumor regression while minimizing postoperative complications.

### WES Library Preparation and Sequencing

Tumor RNA was extracted from pre‐ and post‐nCRT rectal tissue samples. RNA libraries were prepared using the Illumina Whole Exome Sequencing (IWES) protocol according to the manufacturer's instructions. Paired‐end sequencing was performed on the Illumina NovaSeq platform to generate high‐quality transcriptome data.

### Variant Calling and Filtering

WES reads were aligned to the human reference genome GRCh38 using BWA‐MEM (version 0.7.17),^[^ ^69^ ^]^ followed by preprocessing steps, including duplicate marking, base recalibration, somatic mutation calling, and mutation filtering using the Genome Analysis Toolkit (GATK; version 4.4.0.0) based on default parameters. ANNOVAR was then applied to annotate the rest variants. Further variation analysis and visualization were applied using the R package “maftools” (version 2.18.0), including tumor mutation burden analysis and calculation of the number and proportion of SNP transitions/transversions.

### Microsatellite Analysis

MSIsensor2(version 1.3) was applied to detect the MSI status and calculate the MSI score (number of MSI sites/all valid sites) of the samples. MSI data of TCGA‐READ was obtained from a previous study,^[^ ^70^ ^]^ and the samples were classified as MSS or MSI based on MSI scores. An MSI score = 3.5 was set as a cutoff to distinguish MSS and MSI.

### Copy Number Variance Analysis

Cnvkit (version 0.9.12) was used to examine the cnv status of each sample. First, the per‐sample CNV profiles were generated using the cnvkit.py batch command with the aligned BAM files as input and producing a cnr (Copy Number Ratio) file and a cns (Copy Number Segments) file for each sample. Subsequently, gene‐level CNV information was obtained using cnvkit.py genemetrics, which maps the segmented CNV data to gene coordinates and calculates copy number metrics for each gene.

### Bulk RNA‐Seq Library Preparation, Sequencing, and Pre‐Processing

Total RNA was extracted from tumor tissues using TRIzol reagent. Bulk RNA‐seq libraries were prepared with the Illumina TruSeq RNA Library Prep Kit and sequenced on the NovaSeq 6000 platform to generate 150 bp paired‐end reads. Raw data were quality‐checked and trimmed using FastQC and Trimmomatic.

### Single Cell RNA‐Seq Library Preparation and Sequencing

Single‐cell suspensions were prepared from tumor tissues and processed using the 10x Genomics Chromium platform. Libraries were constructed following the Single Cell 3′ v3.1 protocol and sequenced on an Illumina NovaSeq 6000 platform to obtain paired‐end reads for downstream transcriptomic analysis.

### Single Cell RNA‐Seq Data Pre‐Processing and Cell Clustering

Raw sequencing reads were aligned to the human reference genome (GRCh38) using the CellRanger toolkit (version 7.1.0). And cellranger count function was used to generate the gene‐cell unique molecular identifier (UMI) matrix. Scrublet (version 0.2.3)^[^ ^71^ ^]^ was applied to identify potential doublets in each sample, which were removed in further analysis, and Seurat (version 4.3.0)^[^ ^72^ ^]^ was used to pre‐process and cluster the remaining cells. To ensure high‐quality datasets, the percentage of counts originating from mitochondrial RNA was calculated. Cells of low quality were excluded based on the following criteria: 1) mitochondrial gene expression exceeding 5 median absolute deviations (MADs) above the median, 2) UMI count deviating by more than 3 MADs from the median, and 3) gene count deviating by more than 3 MADs from the median.

Subsequently, “NormalizeData” and “FindVariableFeatures” were applied to normalize and generate the 2000 most variable genes for each sample. To integrate cells across different samples, “IntegrateData” was used to correct batch effects and harmonize data integration based on repeatedly variable features across samples. After performing “RunPCA” and “FindNeighbors” on the top 50 principal components (PCs), cell clustering was conducted using “FindClusters”. Resolution parameter resolution = 2.5 was applied during clustering. And the broad cell types were annotated by well‐established cell markers.

### Cell Type Annotation

To identify major cell types, T cells were annotated based on the expression of *CD3D*, *CD3E*, *CD2*, and *TRBC1*; B cells were identified by the expression of *CD79A*, *CD79B*, and *MZB1*; and myeloid cells were marked by *CD14* and *CD68*. For stromal cells, fibroblasts were distinguished by the expression of *ACTA2*, *COL3A1*, and *DCN*, while endothelial cells were annotated by the high expression of *PECAM1* and *VWF*. Epithelial cells were identified by the co‐expression of *EPCAM* and *CEACAM5*. Enteric glial cells were annotated using *NRXN1* and *SOX10* (Figure S2b, Supporting Information).

T cells were further categorized into CD8+ and CD4+ subsets based on the expression of *CD8A*, *CD8B*, and *CD4*. Within the CD8+ T cell subset, markers such as *XCL1*, *XCL2*, *GZMK*, *CMC1*, *HLA‐DRB1*, *GZMH*, *TOX*, *TIGIT*, *MKI67*, and *TOP2A* were used to identify resident memory T cells (Trm), early effector memory T cells (Tem), Tem, exhaustion T cells (Tex), proliferative T cells, and intraepithelial lymphocytes (IELs). For CD4+ T cells, naive T cells (Tn), follicular helper T cells (Tfh), helper T cells (Th), and regulatory T cells (Tumor‐Treg) were identified by the expression of *CCR7*, *SELL*, *LEF1*, *ICA1*, *TOX2*, *IL7R*, *CCR6*, *CD40LG*, *FOXP3*, and *CTLA4* (Figure S4a, Supporting Information). For B cells, CD20+ B cells and plasma cells were distinguished by *MS4A1* and *XBP1*, while sub‐clusters including naive B cells (Bn), memory B cells (Bmem), germinal center B cells (Bgc), IgA plasma cells, and IgG plasma cells were identified based on the expression of *FCER2*, *TCL1A*, *IGHD*, *CD27*, *AIM2*, *TNFRSF13B*, *RGS13*, *LMO2*, *NEIL1*, *BCL6*, *IGHA1*, *IGHA2*, *IGHG1*, and *IGHG2* (Figure S6b, Supporting Information). As for myeloid cells, mast cells were identified by the expression of *CPA3* and *KIT*; macrophages were marked by *CD68* and *CD163*; dendritic cells were annotated by *CD14*, *HLA‐DRB1*; and neutrophils were distinguished based on *FCGR3B* and *CSF3R*, with sub‐clusters named by specific expressed genes (Figure S4b, Supporting Information). For fibroblast cells, adipose fibroblasts (CAF_adi) were identified by the expression of *CFD*, *FBLN1*, *GSN*, and *IGFBP6*; antigen‐presenting fibroblasts (CAF_ap) were identified by *HLA‐DRA*, *LYZ*, and *HLA‐DRB1*; inflammatory fibroblasts (CAF_inflam) were distinguished by the expression of *PDGFRA*, *TGFB1*, and *POSTN*, while myofibroblast‐like fibroblasts (CAF_myo) were annotated using *ACTA2*, *MYH11*, and *RGS5* (Figure S3b, Supporting Information). For endothelial cells, capillary endothelial cells (Capillary_EC HSPH1+), tip‐like endothelial cells (Tip‐like_EC PLVAP+), venous endothelial cells (Venous_EC HLA‐DRA+), arterial (Arterial_EC CXCL12+), and lymphatic endothelial cells (Lymphatic_EC LYVE+) were identified by expression of *HSPH1*, *CEBPB*, *PLVAP*, *COL4A1*, *ACVR1*, *SELP*, *SLC2A1*, *VCAM1*, *SELP*, *VCAM1*, *SEMA3G*, *IGFBP3*, *CXCL12*, *LYVE1*, *PROX1*, *TFF3* (Figure S6c, Supporting Information).

### Single‐Cell Copy Number Variation Analysis

In order to distinguish malignant cells from normal epithelial cells, we used copyKAT (version 1.1.0)^[^ ^73^ ^]^ to infer copy number variation (CNV) of each epithelial cell. The UMI count matrix of all epithelial cells was used as input; the software first processes and clusters the UMI data, selecting diploid cells with high confidence. Hierarchical clustering is then applied to identify aneuploid cells that significantly differ from diploid cells. The calculated copy number matrix and prediction results were retained for further analysis. Predicted aneuploid cells are inferred as tumor cells; diploid cells are stromal normal cells.

### Differential Expression and Functional Annotation

Differentially expressed genes (DEGs) were identified by the “FindAllMarkers” function from the Seurat (version 4.3.0)^[^ ^72^ ^]^ package. Genes with a log2Fold change >0.5 and adjusted *p*‐value< 0.05 were considered significant DEGs. Gene Ontology analyses of DEGs were carried out using the clusterProfiler (version 4.6.2)^[^ ^74^ ^]^ package. The org.Hs.eg.db (version 3.16.0) annotation database was used to map gene identifiers.

### Gene Signature Calculation

Gene sets related to T cell effector, exhaustion, and genes associated with “‘classically activated”’ (M1) macrophages and “‘alternatively activated”’ (M2) macrophages, progenitor exhaustion and terminal exhaustion signatures, were gathered from previous studies.^[^ ^37^, ^38^, ^45^, ^46^, ^47^, ^48^ ^]^ DC signatures, including “Activation”, “Apoptosis”, “Immune suppressive” and “migration”, and exhaustion signatures for Tfh were also obtained from previous studies^[^ ^64^, ^65^, ^75^, ^76^, ^77^ ^]^ (Table S2, Supporting Information). Specific gene signatures of DC LAMP3+ were defined as the top 20 DEGs ranked by log‐transformed ratio of the normalized gene expression in DC LAMP3+ relative to other DCs(log2_avg) (Table S4, Supporting Information). To identify neoCRT‐related TLS signatures (Table S3, Supporting Information), TLS‐related gene signatures identified in previous studies^[^ ^54^, ^59^, ^60^, ^61^ ^]^ were collected, and genes that were significantly differentially expressed before and after treatment were kept.

Gene set variation analysis (GSVA) was conducted using the GSVA package (version 1.46.0).^[^ ^78^ ^]^ The gene sets employed for the analysis were sourced from the MSigDB (version 7.5.1). To compare pathway activity scores between TAM‐MRC1+ and TAM‐VEGFA+, as well as between early‐Tex and terminal‐Tex cells (Figure 3B), differential analysis was performed using the limma package (version 3.54.2).^[^ ^79^ ^]^ fgsea package (version 1.24.0)^[^ ^80^ ^]^ was used for applying Gene Set Enrichment analysis(GSEA).

Signature scores calculated using “AddModuleScore” in Seurat. The *p*‐values were calculated using wilcox.test. And the comparison of progenitor exhaustion and terminal exhaustion signatures was performed by Vision (version 3.0.2).^[^ ^81^ ^]^

### Cell‐To‐Cell Communication Analysis

CellChat(version 1.6.1)^[^ ^82^ ^]^ was used to explore cell‐to‐cell interactions between cell clusters. In order to identify the changes of the cell–cell communication networks related to neoCRT, the database CellChatDB.human was used to identify overexpressed ligands and receptors, and group cells according to their cell type and clinical group: responder pre, responder post, non‐responder pre‐, and non‐responder post. A vignette of comparison analysis of multiple datasets with different cell type compositions was then followed in the CellChat github repository (https://github.com/jinworks/CellChat). To facilitate the visualization of up‐regulated and down‐regulated signaling ligand‐receptor pairs, the difference in communication possibility before and after treatment based on the results of the “netVisual_bubble” function was calculated.

### Cell Developmental Trajectory

Development trajectories of CD8+ T subsets (CD8+ early Tem, CD8+ Tem, CD8+ Trm, CD8+ Tex), CD4+ T subsets(CD4+ Tn, CD4+ Th, CD4+ Tfh, Tumor‐treg), CAF subsets(CAF_myo ACTA2+, CAF_adi CFD+, CAF_infla POSTIN+ and CAF_ap HLA‐DRA+), TAM subsets (TAM MKI67+, TAM APOE+, TAM CXCL1+, TAM MRC1+ and TAM VEGFA+), DC subsets (DC_BATF3+, DC_FCER1A+ and DC_LAMP3+) and TLSs were inferred by using Monocle(version 2.26.0).^[^ ^83^, ^84^ ^]^ The “differentialGeneTest” function was applied to calculate DEGs of each cell subtype, and genes with qval< 0.01 were used to order cells in further analysis. “BEAM” was used to identify genes with branch‐dependent expression.

### Gene Regulatory Network Analysis

In order to identify cell‐type‐specific TFs with direct target genes (regulons), single‐cell regulatory network inference and clustering (SCENIC) analysis was conducted using pySCENIC (version 0.12.1).^[^ ^67^ ^]^ The raw count matrix from all samples was used as input, and all parameters were set to their default values. The analysis followed a three‐step process: 1) The “pyscenic grn” function was applied to calculate co‐expression modules and assess the relationships between transcription factors (TFs) and their target genes; 2) pyscenic ctx was used to identify regulons; 3) “pyscenic aucell” was used to identify the activity of each regulon in individual cells. For visualization, average regulon activity (AUC) scores were calculated for each cell type, and a rank plot of regulons was generated using ggplot2. hdWGCNA was utilized to identify genes that are positively regulated by NR1D2. Initially, the “MotifScan” function was employed to search for instances of various transcription factor (TF) motifs within the promoter regions of genes. Subsequently, the “ConstructTFNetwork” function was used to build a network connecting TFs and their potential target genes. Based on the inferred relationships within the network, genes that were positively correlated with *NR1D2* were considered as the *NR1D2* positively regulated genes.

### TLS Identification and Integrative Analysis

For two single‐cell spatial transcriptome samples (ST‐CR1 and ST‐NR1), the average gene expression and spatial coordinates of each bin were used as inputs to construct the Seurat object using Seurat. To identify TLS in each sample, a gene score for each ST bin was first calculated by summing the expression of signature genes for T cells and B cells. Thresholds were then set based on the corresponding single‐cell data: T‐cell scores in the top 23.9%, B‐cell scores in the top 5.4%. Bins with T‐cell and B‐cell scores above their respective thresholds were defined as TLS regions. TLSs from CR1 and NR1 were further integrated together using stlearn(0.4.12)^[^ ^85^ ^]^ and harmonypy(0.0.10).

### ROC Analysis

To assess the diagnostic power of the TLS signatures, ROC (Receiver Operating Characteristic) curve analysis was performed using the pROC package(version 1.18.5).^[^ ^86^ ^]^ A ROC curve was generated for the TLS signatures, based on the summed expression scores of the gene set. The Area Under the Curve (AUC) values for the gene set were calculated and reported to evaluate the ability to distinguish TLS from no‐TLS cells.

### Correlation Analysis

Pearson correlation coefficients were calculated to assess the relationships between DC_LAMP3+ marker expression and various signatures, including Tfh exhaustion, TLS signature expression, as well as the correlation between IRF8 regulon expression and DC activation and immune suppression pathway.

### Multiplex Immunohistochemistry Analysis

Multiplex immunohistochemistry was performed on formalin‐fixed, paraffin‐embedded (FFPE) tumor sections using the Opal Polaris 7‐color IHC kit (Akoya Biosciences) following the manufacturer's protocol. Slides were scanned with the Vectra Polaris system and analyzed using inForm software.

### Statistical Analyses

The Wilcoxon rank‐sum test was used to compare gene expression between two distinct cell groups, with a *p*‐value< 0.05 considered statistically significant. Differentially enriched gene sets were determined using a two‐sided unpaired limma‐moderated *t*‐test, and a *p*‐value< 0.05 was considered statistically significant. A tailed *t*‐test was used to compare the abundance of early‐Tex in responders and non‐responders.

### Ethics Approval Statement

This study was conducted in accordance with the Declaration of Helsinki. The Ethics Committee of Tongji Hospital, Shanghai (Approval No. 2023–034), and the study was registered in the Chinese Clinical Trial Registry (ChiCTR2300070508).

## Conflict of Interest

The authors declare no conflict of interest.

## Author Contributions

M.W., J.S., K.X., L.Y., and X.Y. contributed equally to this work. J.S. and X.L. conceived and developed the outline of this research and J.S. is the lead contact of this work. M.W. and L.Y. performed the data analysis and method evaluations. K.X., X.Y., and Q.H. performed patient sample collection and cell experiments with the help of J.W., L.Z., X.Y., J.Q., W.W., L.C.Z., X.D., Z.Z., J.W., D.L. and Z.H. M.W., J.S., and X.L. wrote the paper with the help of all other authors.

## Supporting information



Supporting Information

## Data Availability

The raw sequencing data generated in this study have been deposited in the Genome Sequence Archive (GSA)^[^ ^87^ ^]^ at the National Genomics Data Center (NGDC),^[^ ^88^ ^]^ China National Center for Bioinformation / Beijing Institute of Genomics, Chinese Academy of Sciences, under accession number GSA‐Human: HRA011477. These data are publicly available at: https://ngdc.cncb.ac.cn/gsa‐human and available on request from the corresponding authors. The spatial transcriptomics data used in this study can be obtained from the China National GeneBank Database(CNGBdb) with accession number CNP0004138.
