# Supplementary material for: T Cell Exhaustion and Dendritic Cell‐Mediated Tertiary Lymphoid Structures (TLSs) Modulation Affect Response to Neoadjuvant Chemoradiotherapy in Microsatellite Stable Rectal Cancer
Source: Adv Sci (Weinh). 2025 Nov 5;13(4):e14332. doi: 10.1002/advs.202514332 (PMC12822459; doi:10.1002/advs.202514332)
Supplement: Supplementary file 1 — Supporting Information [file ADVS-13-e14332-s001.pdf]

Supporting Information

T Cell Exhaustion and Dendritic Cell-Mediated tertiary lymphoid structures (TLSs) Modulation Affect Response to Neoadjuvant Chemoradiotherapy in Microsatellite Stable Rectal Cancer

Miao Wang, Jiejun Shi, Kai Xu, Leyi Yu, Xiaomao Yin, Jiexuan Wang, Lin Zhu, Xin Yang, Jingjing Qian, Wenqiang Wang, Liangchen Zhu, Xuan Dai, Zekun Zhao, Jinran Wu, Dongsheng Li, Zhiqian Hu, Qi Huang*, Xinxing Li**

Supplementary Figures

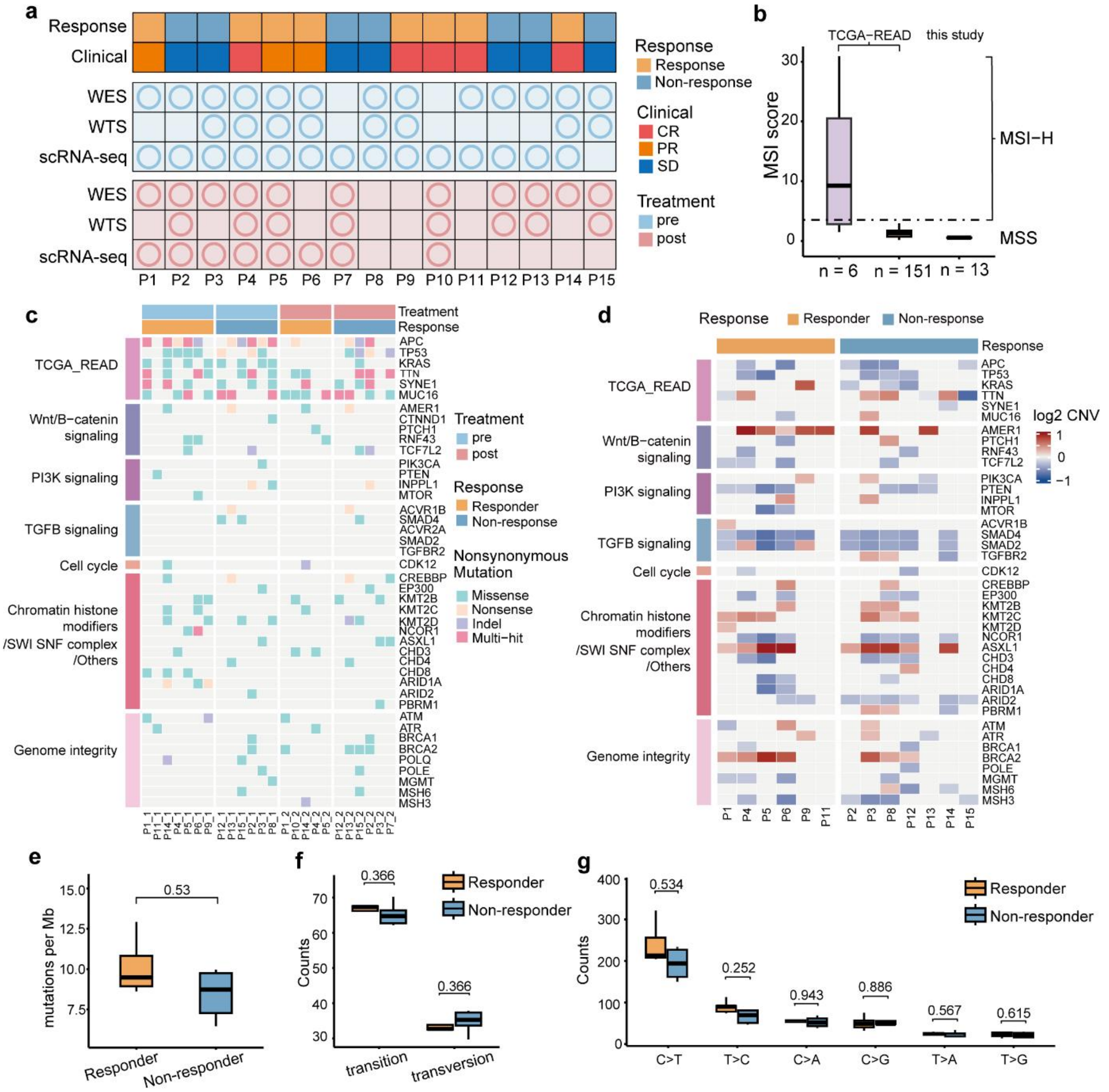

Figure S1. Somatic mutational landscape of study samples

a Clinical outcomes and sample collection details for each patient. **b** Microsatellite instability (MSI) scores of TCGA samples and samples from this study. A cutoff of MSI score = 3.5 was used. Samples with MSI scores > 3.5 were classified as MSI-H (high microsatellite instability), while those with scores ≤ 3.5 were classified as MSS (microsatellite stable). **c** Mutation landscape of LARC patients pre- and post-neoCRT. **d** Copy number variations (CNVs) in key cancer-related pathways stratified by treatment response. The color scale denotes log2 CNV values compared to (red: amplification; blue: deletion). **e** Tumor mutational burden (TMB) per megabase (Mb) in responders versus non-responders. Statistical significance was determined using the Wilcoxon test. **f** and **g** Counts of transition and

transversion (f) and single nucleotide variants (SNVs) (g) detected in responders versus non-responders. Statistical significance was determined using the Wilcoxon test.

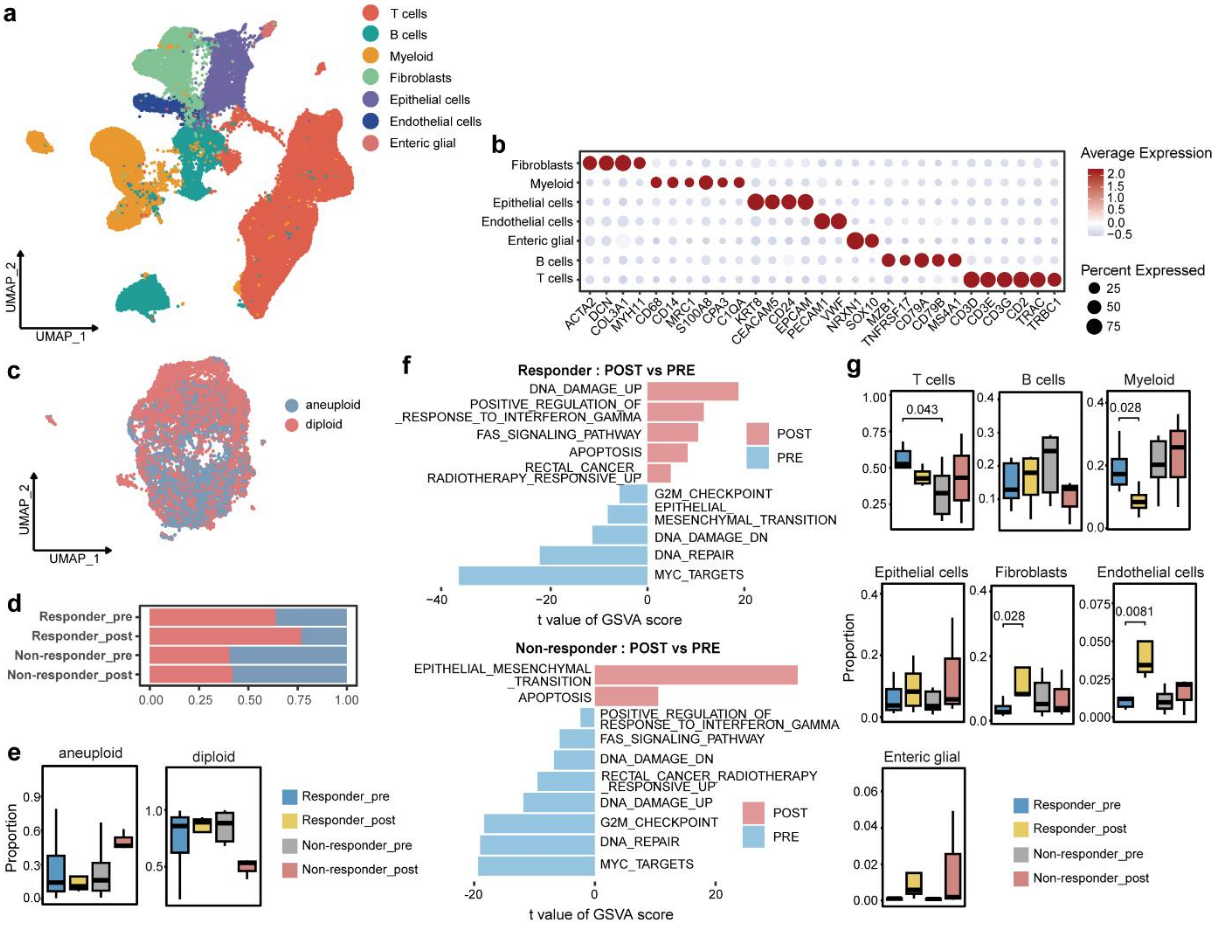

Figure S2. neoCRT damaged DNA to eradicate or control the proliferation of cancer cells

a UMAP of all cells. **b** Expression of marker genes of major cell types. **c** UMAP of epithelial cells. **d** Relative proportions of cell subtypes in pre-treatment and post-treatment samples from responders and non-responders. **e** Boxplots of cellular fractions of tumor cells (aneuploid) and normal cells (diploid) in pre-treatment and post-treatment samples from responders and non-responders. Statistical significance was determined using the Wilcoxon test, and the p values < 0.05 are indicated. **f** Differential pathways enriched in post-treatment versus pre-treatment samples in responders (top) and non-responders (bottom), analyzed by Gene Set Variation Analysis (GSVA). Significance was determined using a two-sided unpaired limma-moderated t-test (p-value < 0.05). **g** Boxplots of cellular fractions of T cells, B cells, myeloid cells, Epithelial cells, Fibroblasts, Endothelial cells and enteric glial cells in pre-treatment and post-treatment samples from responders and non-responders. Statistical significance was determined using the Wilcoxon test, and the p values < 0.05 are indicated.

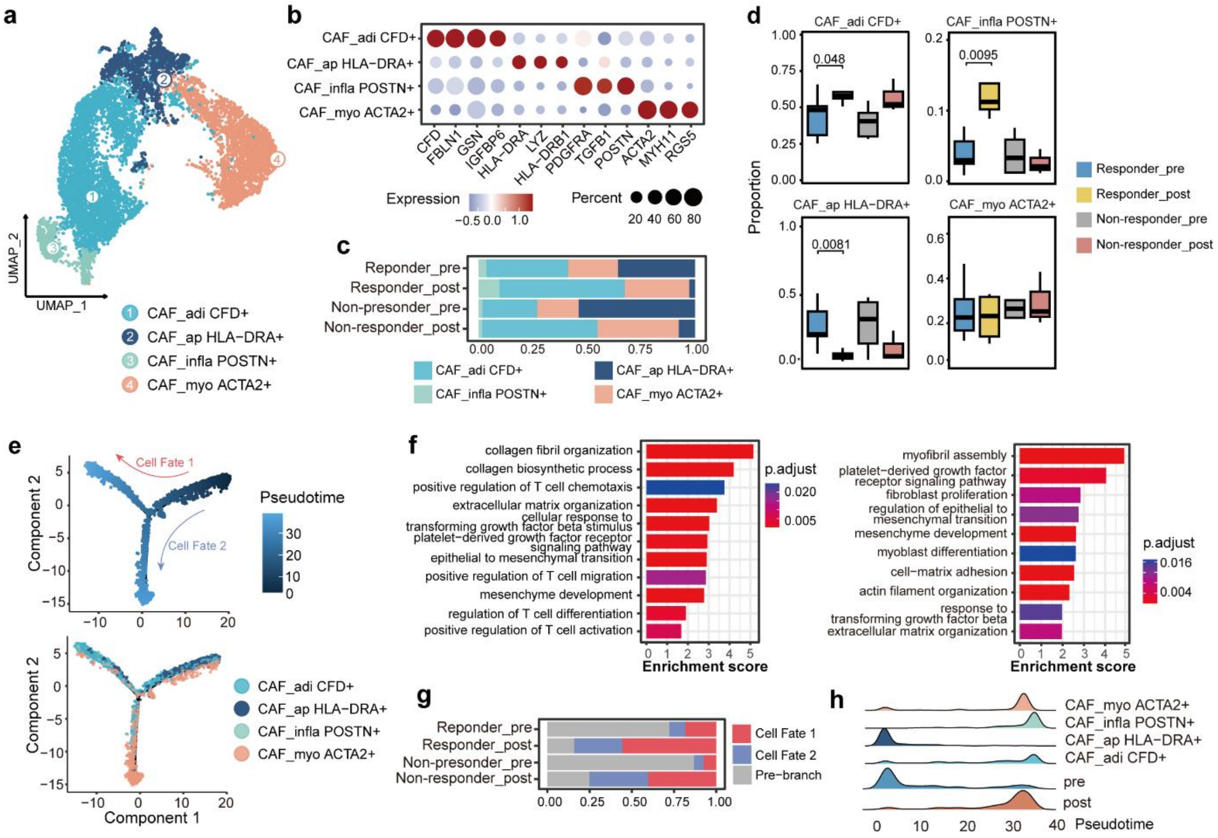

Figure S3. neoCRT induced CAF differentiation and promoted fibrosis

a UMAP of sub clusters of Fibroblasts. b Expression of marker genes of each CAF sub cell-type. **c** Relative proportions of cell subtypes in pre-treatment and post-treatment samples from responders and non-responders. **d** Boxplots of cellular fractions of CAF sub cell-types in pre-treatment and post-treatment samples from responders and non-responders. Statistical significance was determined using the Wilcoxon test, and the p values < 0.05 are indicated. **e** Developmental trajectory of CAFs. Colored by pseudotime (top) and sub-cell types (bottom). Arrows in the top plot denote potential differentiation paths. **f** GO terms enriched in genes up-regulated along with cell fate 1(left) and cell fate 2(right) of CAFs developmental trajectory. Benjamini-Hochberg adjusted p-value<0.05. **g** Relative proportion of cells located at pre-branch, cell fate1 and cell fate2 of CAFs developmental trajectory of pre-treatment and post-treatment samples. **h** Cell density of CAF subsets along the pseudotime.

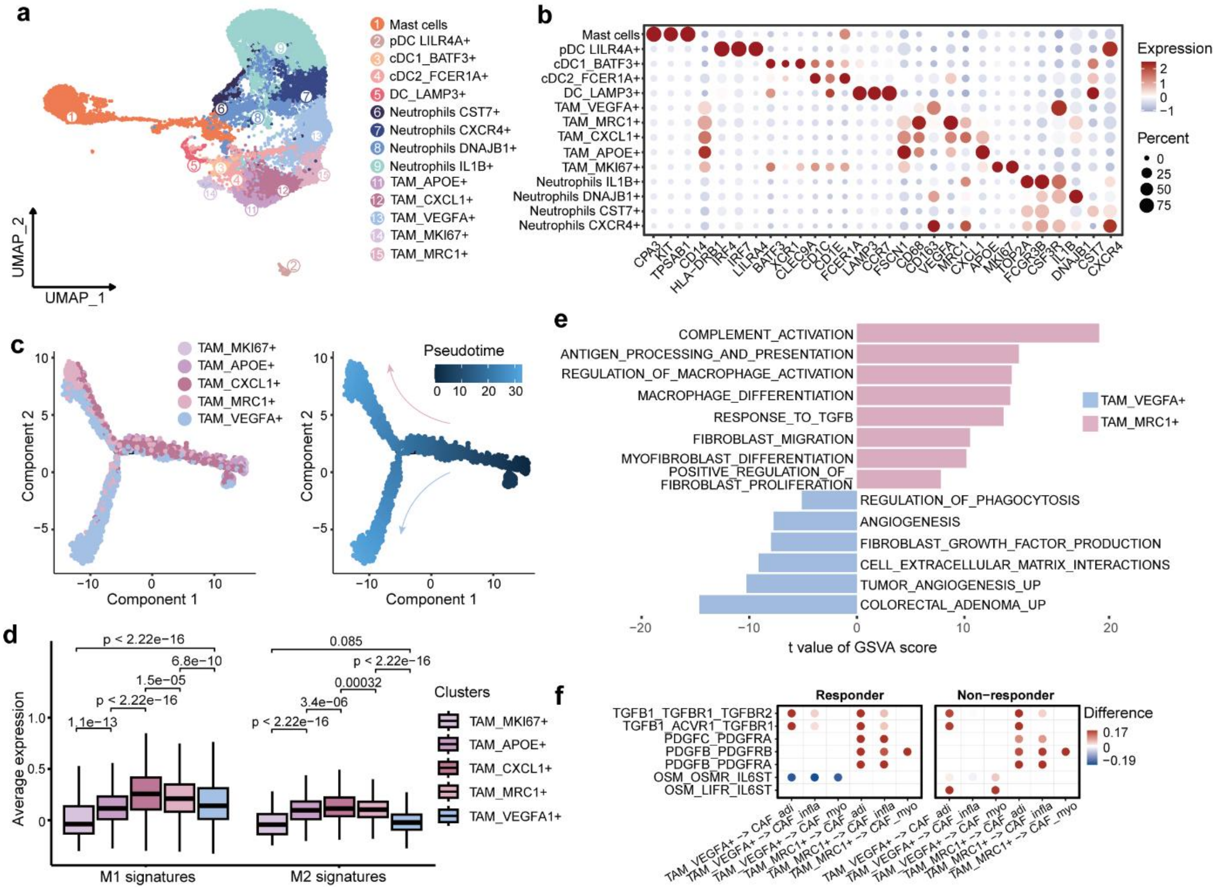

Figure S4. TAMs involved in the activation of CAFs and the promotion of fibrosis

a UMAP of sub-clusters of myeloid cells. **b** Expression of marker genes of each myeloid sub cell-type. **c** Developmental trajectory of TAMs. Colored by cell types(left) and pseudotime (right). Arrows in the right plot denote potential differentiation paths. **d** M1 and M2 signature scores across TAM subsets. Statistical significance was determined using the Wilcoxon test. **e** Differential pathways enriched in MRC1+ and VEGFA+ TAMs, analyzed by Gene Set Variation Analysis (GSVA). Significance was determined using a two-sided unpaired limma-moderated t-test (p-value < 0.05). **f** Altered ligand-receptor (L-R) pair signaling from TAM MRC1+ and TAM VEGFA+ to T cells. Dot colors indicate the difference in communication probability between post-treatment and pre-treatment.

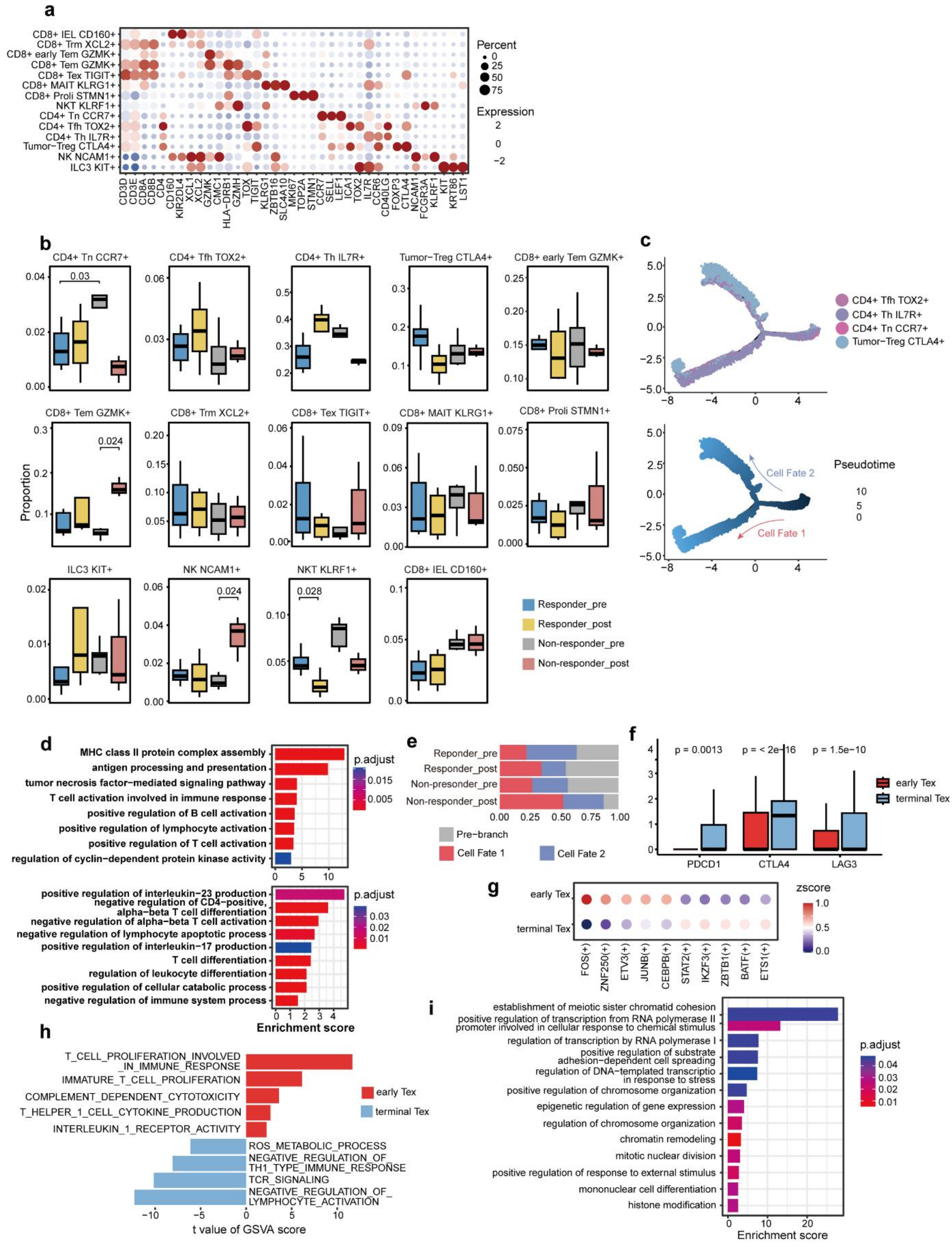

Figure S5. CD4+ T cells differentiate into two branches and NR1D2 plays an important role in early-Tex

a Expression of marker genes of each T cells sub cell-type. **b** Boxplots of cellular fractions of T cells sub cell-types in pre-treatment and post-treatment samples from responders and non-responders. Statistical significance was determined using the Wilcoxon test, and the *p*

values < 0.05 are indicated. **c** Developmental trajectory of CD4+ T cells. Colored by cell types(top) and pseudotime (bottom). Arrows in the right plot denote potential differentiation paths. **d** GO terms enriched in genes upregulated along cell fate 1 (top) and cell fate 2 (bottom) of the CD4+ T cell developmental trajectory. Benjamini-Hochberg adjusted p-value<0.05. **e** Relative proportion of cells located at pre-branch, cell fate1 and cell fate2 in pre-treatment and post-treatment samples. **f** Expression of co-inhibitor genes between early and terminal-Tex. p-values were calculated by Wilcox.test. **g** Specific TF regulons identified in early and terminal-Tex. **h** Differential pathways enriched in early-Tex versus terminal-Tex, analyzed by Gene Set Variation Analysis (GSVA). Significance was determined using a two-sided unpaired limma-moderated t-test (p-value < 0.05). **i** GO terms enriched in NR1D2 target genes. Benjamini-Hochberg adjusted p-value<0.05.

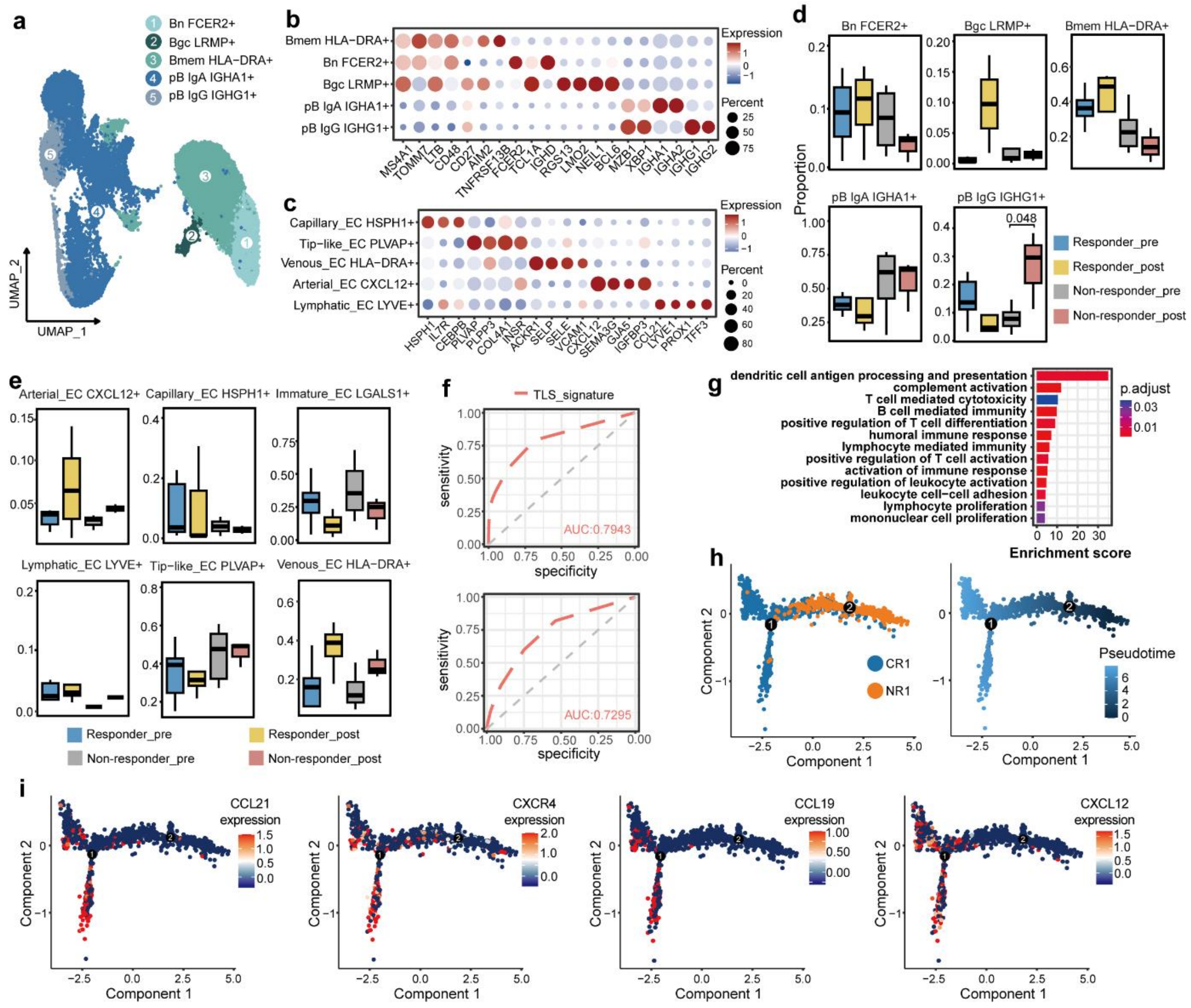

Figure S6. neoCRT promotes the formation of more activated and mature TLSs in responders

a UMAP of sub-clusters of B cells. **b** Expression of marker genes of each B cell sub cell-type. **c** Expression of marker genes of each endothelial cell sub cell-type. **d** Boxplots of cellular fractions of B cells sub cell-types in pre-treatment and post-treatment samples from responders and non-responders. Statistical significance was determined using the Wilcoxon test, and the p values < 0.05 are indicated. **e** Boxplots of cellular fractions of CAF sub cell-types in pre-treatment and post-treatment samples from responders and non-responders. Statistical significance was determined using the Wilcoxon test, and the p values < 0.05 are indicated. **f** ROC curves for TLS signatures prediction of TLS in CR and NR. **g** GO terms enriched in genes upregulated in TLSs of responders compared to non-responders. Benjamini-Hochberg adjusted p-value<0.05. **h** Developmental trajectory of TLSs. Colored by samples(left) and pseudotime (right). **i** Expression of TLS activation- and maturation- related genes along the developmental trajectory.

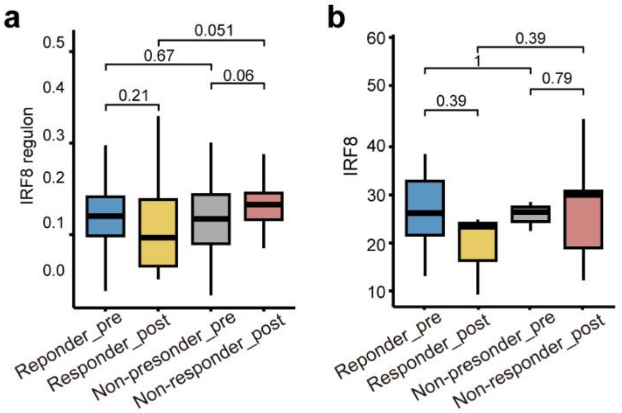

Figure S7. Expression of IRF8-regulon and IRF8 gene

a Expression of IRF8 regulon across four groups in single-cell RNA-seq data. *p*-values were calculated by Wilcox.test. **b** Expression of IRF8 gene across four groups in bulk RNA-seq data. *p*-values were calculated by Wilcox.test.

Supplementary Tables

Patient	Gender	Age	Primary Diagnosis	Pathology Type	Distant Metastasi	Neoadjuvant Chemotherapy	Neoadjuvant Radiotherapy	Radiological Tumor Response
P1	Male	71	Rectal cancer	Adenocarcinoma	No	Oxaliplatin+Capecitabine	Yes	PR
P2	Male	68	Rectal cancer	Adenocarcinoma	No	Oxaliplatin+Capecitabine	Yes	SD
P3	Female	52	Rectal cancer	Adenocarcinoma	No	Oxaliplatin+Capecitabine	Yes	SD
P4	Female	40	Rectal cancer	Adenocarcinoma	No	Oxaliplatin+Capecitabine	No	CR
P5	Male	65	Rectal cancer	Adenocarcinoma	No	Oxaliplatin+Capecitabine	No	PR
P6	Male	62	Rectal cancer	Adenocarcinoma	No	Oxaliplatin+Capecitabine	Yes	PR
P7	Male	47	Rectal cancer	Adenocarcinoma	No	ACRnaCT	Yes	SD
P8	Male	53	Rectal cancer	Adenocarcinoma	No	Oxaliplatin+Capecitabine	Yes	SD
P9	Female	54	Rectal cancer	Adenocarcinoma	No	ACRnaCT	Yes	CR
P10	Female	57	Rectal cancer	Adenocarcinoma	No	Oxaliplatin+Capecitabine	Yes	CR
P11	Female	76	Rectal cancer	Adenocarcinoma	No	Capecitabine	Yes	CR
P12	Male	49	Rectal cancer	Adenocarcinoma	No	Oxaliplatin+Capecitabine	No	SD
P13	Female	58	Rectal cancer	Adenocarcinoma (mostly mucinous	No	Oxaliplatin+Capecitabine	Yes	SD
P14	Male	62	Rectal cancer	Adenocarcinoma	No	Oxaliplatin+Capecitabine	No	CR
P15	Male	71	Rectal cancer	Mucinous adenocarcinoma	No	Oxaliplatin+Capecitabine	No	SD

Table S1. Clinical Characteristics and Treatment Response of Locally Advanced Rectal Cancer Patients

Maccrphage		T cell				Dendritic cell			
M1	M2	Exhaustion	Progenitor exhaustion	Terminal exhaustion		Activation	Immune suppressive	Apoptosis	Migration
CCL3	CD163	CXCL13	IL7R	NKG7	XRCC6	FSCN1	IDO1	AKT3	GAL3ST
CCL4	ARG2	HAVCR2	GPR183	RAC2	C17orf62	BIRC3	CCR7	IRAK3	NUDT17
CCL2	IL4R	PDCD1	LMNA	CLIC1	PRR13	LAMP3	LGALS3	CHP1	ITGB8
IFNG	CCL13	TIGIT	NR4A3	GZMA	HLA-DRB5	CCL19	LGALS9	CHUK	ADCY6
IRF5	CCL17	LAG3	TCF7	PRF1	WDR1	LAD1	NECTIN2	CSF2RB	ENO2
IRF1	CCL18	CTLA4	MGAT4A	APOBEC3C	ARL6IP1	MARCKS	CD274	DFFA	IL15RA
IL23A	CCL22	LAYN	CD55	RHOA	ISG15	TNFAIP2	PDCD1LG2	DFFB	SOCS2
TNF	CCL24	RBPJ	AIM1	CCL4	ATP5A1	CCR7		ENDOG	IL15
KYNU	LYVE1	VCAM1	PER1	COTL1	EWSR1	CCL22		AKT1	STAP2
IL6	VEGFA	GZMB	FOSL2	PSME2	COPE	MARCKSL1		AKT2	PHF24
CD40	VEGFB	TOX	EGR1	HLA-DPA1	HAVCR2	EBI3		ENDOD1	ANKRD33B
CXCL9	VEGFC	MYO7A	TSPYL2	HMGN2	EIF3H	TNFRSF11B		PIK3R5	INSM1
CXCL10	VEGFD		YPEL5	LSP1	ANXA5	NUB1		APAF1	ANXA3
CXCL11	EGF		CSRNP1	PSMB9	C11orf58	INSM1		BIRC2	ARHGAP28
CCL5	CTSA		REL	LCK	IFI6	RAB9A		BIRC3	RNF115
CCR7	CTSB		SKIL	SRP14	SIRPG	LY75		XIAP	ADORA2A
IL1A	CTSC		PIK3R1	ARPC3	CALM3	SIAH2		FAS	EXTL1
IL1B	CTSD		FOXP1	ARPC1B	SHISA5	POGLUT1		IKBKB	SPSB
CD86	TGFB1		RGCC	TPI1	DENND2D	KDM2B		IL1A	SLC22A23
CD80	TGFB2		PFKFB3	APOBEC3G	MAP4K1	MGLL		IL1B	RABGAP1
CD68	TGFB3		MYADM	HLA-DPB1	BUB3	TXN		IL1R1	GYG1
NOS2	MMP14		ZFP36L2	LDHB	IKZF3	MLLT6		IL1RAP	DAP
HLA-DPB1	MMP19		USP36	ATP5G2	SNRPB	KIF2A		FASLG	OGFR
MARCO	MMP9		TC2N	MYL12B	EID1	GRSF1		IL3	GYG2
IL2RA	CLEC7A		FAM177A1	PSMB8	PSMB1	FAM49A		IL3RA	CCSER2
IL15	WNT7B		BTG2	PSMA7	PTPN6	PLEKHG1		IRAK1	TMEM123
CD14	FASLG		TSC22D2	HLA-DRB1	NDUFA13	SOCS2		IRAK2	NET1
FCGR3A	TNFSF12		FAM65B	SUB1	SSR4	RFTN1		MYD88	GPR52
FCGR1A	TNFSF8		STAT4	ARPC4	COX8A	AC009812.4		ATM	SLCO5A1
FCGR1B	CD276		RGPD5	CTSW	PTPN7	BMP2K		NFKB1	FAH
IDO1	FN1		NEU1	SUMO2	MAT2B	NAV1		NFKBIA	CLU
	IRF4		IFRD1	TAP1	PSTPIP1	IL7R		NGF	PCGF5
	IDO1		PDE4B	GZMB	GSTP1	ID2		NTRK1	SAMSN1
	FABP4		NR4A1	RARRES3	PSMB3	CCL17		IRAK4	CDKN2B
	CCR2			CAP1	IRF9	PPP1R9B		PIK3CA	BMP2K
	CD1B			UCP2	TRAF3IP3	NRP2		PIK3CB	ZC2HC1A
	CD1A			PPIB	GIMAP7	TUBB6		PIK3CD	SERINC5
	ALOX15			RAN	PSMA2	ARNTL2		PIK3CG	HIVEP1
	CCL26			CHCHD2	SASH3	UVRAG		PIK3R1	CNR1
	CHN2			PARK7	CD164	TXNDC11		PIK3R2	CNR2
	CCL4			HCST	ETNK1	MREG		CYCS	
	CCL20			GABARAP	S100A11	BTG1		PPP3CA	
	MSR1			HLA-DRA	KLRD1			PPP3CB	
				SOD1	MOB1A			PPP3CC	
				CAPZB	SH2D1A			PPP3R1	
				S100A4	UBE2V1			PPP3R2	
				RNASEK	SH3KBP1			PRKACA	
				PPP1CA	ATP5G3			PRKACB	
				PKM	PSMA5			PRKACG	
				IFI16	MT2A			PRKAR1A	
				ACTR3	LAT			PRKAR1B	
				ITM2A	IFNG			PRKAR2A	
				SLC25A5	RAB27A			PRKAR2B	
				PGAM1	COX5A			PRKX	
				ANXA6	DDOST			BAD	
				CD27	PSMB4			BAX	
				ATP5B	SRP9			BCL2	
				LYST	BRK1			RELA	
				PSMB10	TNFRSF1B			BCL2L1	
				MIF	EIF4H			BID	
				LY6E	GMFG			CHP2	
				ANKRD10	ANXA2			TNF	
				CTSD	TCEB2			TNFRSF1A	
				UBE2L6	RBPJ			TP53	
				EDF1	COX6A1			TRAF2	
				NONO	UBXN1			CAPN1	
				TIGIT	PSMD8			CAPN2	
				FKBP1A	CD63			CASP3	
				IL2RB	ATP6V0E1			CASP6	
				HMGN1	NDUFB8			CASP7	
				ATP5L	CTSC			CASP8	
				GZMH	SNRPD2			CASP9	
				STAT1	ATP5C1			CASP10	
				GPI	PRELID1			PIK3R3	
				LCP2	COX7A2			IKBKG	
				GBP2	PSMA6			TRADD	
				ARL6IP5	ECH1			RIPK1	
				CCL4L1	U2AF1			TNFSF10	
				PRDM1	HMGB2			FADD	
				OST4	FAM49B			TNFRSF10D	
				PDCD1	CD38			TNFRSF10C	
				HINT1	TSPO			TNFRSF10B	
				HNRNPF	IDH2			TNFRSF10A	
				GBP5	CASP4			CFLAR	
				COX7C	CCL3			MAP3K14	
				ARPC5	TRMT112			AIFM1	
				GIMAP4	SURF4			EXOG	
				PRDX1	PSMA1				
				RQCD1	YWHAE				
				CCNDBP1	LASP1				
				INPP4B	PYHIN1				
				CDK2AP2	ANAPC16				
				CBX3	TUBB				
				RPN1	CSNK2B				
				SPCS1	PRKAR1A				
				PSMA3	SLAMF7				
				SIT1	GNG5				
				XRCC5	COX6C				
				EIF3C	M6PR				
				CXCR6	ANP32E				

Table S2. Related gene signatures used for macrophage, T cell and dendritic cell analysis

gene	p_val	avg_log2FC	pct.1	pct.2	p_val_adj	cluster
TXNIP	0.00E+00	1.671760468	0.67	0.452	0.00E+00	post-treatment
CXCR4	0.00E+00	0.985021207	0.782	0.616	0.00E+00	post-treatment
LTB	0.00E+00	0.864901197	0.572	0.428	0.00E+00	pre-treatment
IL7R	8.18E-226	0.810178885	0.557	0.491	2.67E-221	post-treatment
IGLC1	5.75E-27	0.579741271	0.432	0.395	1.87E-22	pre-treatment
TRBC2	6.35E-183	0.5796199	0.59	0.508	2.07E-178	pre-treatment
RAC2	0.00E+00	0.565132692	0.571	0.44	0.00E+00	pre-treatment

Table S3. neoCRT-related TLS signatures

gene	p_val	avg_log2FC	pct.1	pct.2	p_val_adj
CCL19	7.38E-115	3.812983852	0.558	0.015	2.41E-110
FSCN1	4.18E-140	3.466995322	0.841	0.103	1.36E-135
CCL22	2.19E-91	3.337292508	0.558	0.046	7.15E-87
BIRC3	1.23E-77	3.187627137	0.721	0.212	4.00E-73
TXN	1.28E-81	2.982882662	0.946	0.617	4.18E-77
MARCKSL1	2.46E-73	2.889843809	0.795	0.286	8.01E-69
CRIP1	2.97E-72	2.868914017	0.895	0.537	9.68E-68
EBI3	3.10E-80	2.552538241	0.558	0.066	1.01E-75
CCR7	4.15E-74	2.366587642	0.57	0.092	1.35E-69
MARCKS	4.30E-64	2.3640517	0.764	0.29	1.40E-59
TBC1D4	9.49E-82	2.353153259	0.632	0.098	3.09E-77
LAMP3	1.75E-83	2.326294465	0.682	0.129	5.71E-79
CERS6	3.21E-31	2.298670487	0.45	0.158	1.05E-26
NMRK1	7.54E-77	2.139614693	0.465	0.035	2.46E-72
NUB1	6.19E-64	2.133093944	0.64	0.154	2.02E-59

Table S4. Marker genes of DC LAMP3+ (top 15 DEGs vs other DCs)

		LARC Cohort (n=40)
Age, years		69.5 (60.5-73.0)
Sex	Male	23 (57.5%)
	Female	17 (42.5%)
Histological type	Adenocarcinoma	40 (100%)
pathological TNM stage	pStage II	25 (62.5%)
	pStage III	15 (37.5%)
Maximum tumor diameter		5.50 (4.50 -6.80)
Carcinoembryonic Antigen		2.96 (1.98-7.35)

Data are n(%) or median (IQR).

Table S5. Baseline characteristics of population. Categorical variables are presented as n (%), and continuous variables as median (interquartile range, IQR).
